# Chinese expert consensus on imaging examination and diagnosis of nasal cavity and paranasal sinus tumors

**DOI:** 10.3389/fonc.2025.1626584

**Published:** 2025-08-11

**Authors:** Xunan Zhang, Yi Zhang, Lixiao Chen, Yan Sha, Xiaorui Yin, Han Wang, the Radiology Department Association of Hospitals of Shanghai, the Molecular Group of Chinese Society of Radiology, the Medical Imaging Committee of Shanghai Association of Chinese Integrative Medicine

**Affiliations:** ^1^ Department of Radiology, Shanghai General Hospital of Shanghai Jiao Tong University School of Medicine, Shanghai, China; ^2^ Department of Otolaryngology: Head and Neck Surgery, Shanghai General Hospital of Shanghai Jiao Tong University School of Medicine, Shanghai, China; ^3^ Department of Radiology, Eye and Ear Nose Throat (ENT) Hospital of Fudan University, Shanghai, China; ^4^ Shanghai General Hospital Branch of National Center for Translational Medicine (Shanghai), Shanghai, China; ^5^ Jiading Branch of Shanghai General Hospital, Shanghai, China

**Keywords:** nasal cavity, paranasal sinus, CT, MRI, tumors

## Abstract

The complex anatomy and diverse tissue composition of the nasal cavity and paranasal sinuses contribute to a wide variety of tumor pathologies in this region, posing significant diagnostic challenges in clinical practice. Evaluation with computed tomography (CT) and magnetic resonance imaging (MRI) is critical for the diagnosis and management of patients with sinonasal tumors. Radiologists should be proficient in the indications and contraindications for CT and MRI examinations of sinonasal tumors, along with standardized scanning protocols and image quality control requirements. Particular attention must be paid to radiation protection principles for infants, children, and pregnant patients, alongside contrast agent safety guidelines for pregnant and lactating females. Furthermore, radiologists require a thorough understanding of the intricate anatomy of the sinonasal region, the spectrum of common benign and malignant tumor pathologies, and their characteristic imaging manifestations, especially recognizing tumor-specific imaging signs. Finally, adopting a long-term perspective, radiologists should prioritize multidisciplinary collaboration. Integrating clinical practice with emerging technologies-such as multimodal imaging, molecular diagnostics, and artificial intelligence (AI)-is critical for addressing diagnostic complexities, refining therapeutic strategies, and improving patient prognoses. Advancing research in these domains not only strengthens disease management but also deepens the understanding of pathogenesis and treatment response, ultimately enhancing diagnostic accuracy and long-term outcomes.

## Introduction

Imaging of the nasal cavity and paranasal sinuses is a critical component of head and neck radiology, which has gained increasing prominence in recent years. The nasal cavity and paranasal sinuses possess complex and delicate anatomical structures, and are in close proximity to the orbits, oral cavity, nasopharynx, oropharynx, skull base, and other surrounding regions. Consequently, tumors in this area involve multiple medical specialties, including otorhinolaryngology, ophthalmology, stomatology, neurosurgery, general surgery, and vascular surgery. The diverse anatomical components of the nasal cavity and paranasal sinuses lead to a wide range of tumor origins. Furthermore, the clinical presentations of most inflammatory, benign, and malignant tumors in this region lack specificity. Additionally, the incidence of nasal and paranasal sinus tumors is lower compared to other systems, rendering the disease spectrum relatively rare with a limited number of cases. As a result, frontline clinicians, particularly those in large general hospitals, often have an inadequate understanding of these tumors (1). Consequently, the imaging evaluation and diagnosis of nasal and paranasal sinus tumors have remained challenging aspects of clinical diagnosis, teaching, and research.

Conventional X-ray films are limited in evaluating nasal cavity and paranasal sinus tumors due to structural overlap with adjacent maxillofacial regions, orbits, and the skull base, which obscures detailed anatomical visualization. Current imaging primarily employs CT and MRI. CT is adept at delineating fine bony structures, anatomical variants, and osseous erosion, with multi-detector spiral CT providing enhanced spatial resolution through multiplanar reconstructions. MRI offers superior soft tissue contrast, crucial for assessing mucosal changes, fluid retention, polyps, and neurovascular structures, while also aiding in tumor characterization, boundary definition, and extent evaluation. These modalities are complementary in tumor localization, staging, component analysis, treatment monitoring, and recurrence detection. However, in China, standardization of imaging protocols, diagnostic accuracy, and integration into clinical decision-making are suboptimal. To address these issues, the Radiology Department Association of Hospitals of Shanghai, the Molecular Imaging Group of the Chinese Society of Radiology, and the Medical Imaging Committee of Shanghai Association of Chinese Integrative Medicine have developed a consensus guideline. This initiative seeks to optimize imaging workflows, enhance diagnostic precision, reduce errors, improve resource utilization, and support clinical management and research in sinonasal tumor care.

## Contraindications

### CT

High sensitivity to X-rays or unsuitability for X-ray exposure; Patients cannot cooperate with the scan; Iodine contrast agent allergy, hyperthyroidism, and severe heart, liver, or kidney failure cannot undergo enhanced CT scan.

### MRI

Devices containing magnetic materials, such as Cardiac pacemakers, cochlear implants, cardiac stents, artificial heart valve replacement; metallic foreign bodies in the eyes; pregnancy within the first trimester; claustrophobia; metallic dentures affecting imaging quality for nasal and paranasal sinus tumors; patients unable to undergo examinations due to severe illness.

## Imaging examination process

### Non-contrast CT scan

1. Remove metallic jewelry and dentures from the head and face before scan.
2. Spiral scanning is the primary method. It is recommended to use a 64-slice or higher spiral CT.
3. Scanning position: supine, with head fixation via specialized headrest for midline alignment and patient motion restriction.
4. Scanning range: Frontal sinus superior margin to hard palate, with adjustments based on lesion extent while ensuring comprehensive coverage of tumor and sinonasal anatomical structures.
5. Scanning baseline: Lateral positioning film-based with external auditory meatus reference. Baseline: infraorbitomeatal line, supplemented by device-enabled partial-angle scanning for suboptimal patient cooperation.
6. Scanning conditions: Tube voltage: 100 kV-120 kV; Effective tube current: 150 mAs/layer-220 mAs/layer; Collimator width: 10 mm-40 mm; Pitch ≤ 1.0; Matrix: at least 512×512; FOV: 150 mm- 200 mm.
7. Reconstructed slice thickness: The minimum allowable slice thickness is 0.6-0.70 mm.
8. Reconstruction algorithm: Bone algorithm, soft tissue algorithm; Iterative reconstruction algorithm.
9. Reconstruction protocol: Primary reconstruction modality: soft tissue algorithm for sinonasal mass lesions. Optional sagittal plane reconstruction. 3D post-processing for clinical indications. Window parameters: Bone (W1500-3000/C150-400); Soft tissue (W300-400/C40-50).

### Contrast-enhanced CT

1. Examination protocol: Pre-procedural fasting (≥4hr) with informed consent requirement.
2. CE-CT follows non-contrast acquisition via intravenous bolus injection (IV bolus).
3. Contrast parameters: Total iodine load 300-450mgI/kg (weight-adjusted), supplemental saline flush 15-20ml. Contrast injection rate 2.5-3.5ml/s, scan initiation at 50-60s post-injection.

### Non-contrast MRI

1. Scanning range: In principle, the entire tumor should be included. a. Transverse: Anterior cranial fossa (diaphragma sellae level) to the inferior margin of the second cervical vertebra (C2). b. Coronal: Frontal sinus anterior-sphenoid sinus posterior margins. Sagittal: Entire nasal cavity and paranasal sinuses coverage.
2. Coil selection: Head orthogonal coil or head-neck combined coil.
3. Slice parameters: 3.0-5.0mm thickness; 0-1.0mm interval.
4. Sequences: Mandatory: Transverse T1/T2-Weighted imaging (T1/T2WI); Coronal T1WI.Conditional: Sagittal acquisition; fat-sat T2WI for suspected intralesional fat.

### Contrast-enhanced MRI

1. Contrast protocol: Weight-based dosing (0.1mmol/kg); IV rate 1-2ml/s with saline flush.
2. Sequences: Multiplanar T1WI (transverse/coronal/sagittal); DWI with fat-sat on selected planes.
3. Dynamic CE: T1WI acquisitions at 20-30s intervals (10 phases).
4. Parameters: Slice 3-5mm/1mm gap; Matrix ≥224×256; FOV 200×200mm.

## Image quality control

### CT

1.Thin-section bone window radiography: Demonstrates sinonasal osseous alterations (compressive displacement, resorption, infiltrative destruction, cartilaginous matrix presence), aiding tumor origin determination and benign-malignant differentiation.
Artifact control: Reduce metal artifacts by adjusting scanning parameters. Apply iterative reconstruction techniques to reduce noise.
Imaging requirement: Complex sinonasal anatomy necessitates optimal visualization of key structures: cribriform plate; ostiomeatal complex (OMC); pterygopalatine fossa; cranial base foramina.
Ensure the regular calibration and maintenance of equipment.
Contrast administration parameters (dose; flow rate) and ​acquisition timing require precise optimization to prevent injection artifacts (bubble formation; flow irregularity) and suboptimal acquisition timing, ensuring diagnostic image quality.

### MR

1. Fat-sat requirement: Mandatory for skull base/orbital tumor invasion evaluation.
2. Motion control: Head immobilization with controlled respiration; avoidance of swallowing/ocular/lingual activity. Application of sedation protocols for claustrophobic patients.
3. Equipment Quality Control: Scheduled calibration/maintenance protocols.
4. Contrast management: Per established administration standards.

## Examination of special populations

### Children and infants

Special care is needed in imaging children’s nasal and sinus structures due to their small and underdeveloped anatomy. Attention should be given to minimizing radiation dose in CT and controlling motion artifacts in MRI. Additionally, caution is advised regarding the repeated use of contrast agents in a short timeframe due to safety concerns in children and infants (2).

### CT examination

1. Pediatric low-dose CT protocols: Tube voltage 100-120kV and current 100-150mAs/slice are recommended, with age-/weight-adjusted parameter optimization for radiation dose minimization.
2. Contrast administration guidelines: Agent selection: Nonionic isotonic contrast agents; Pediatric dosing: 1.5-2.0ml/kg (weight-based) with three adjustment criteria: weight parameters, manufacturer guidelines, clinical indications; Neonate/preterm protocol: Reduced-dose application principle.
3. Using contrast agents with caution in children with renal insufficiency.
4. Post-procedural care: Guardian-supervised 20-30min observation period; Contrast clearance enhancement via oral hydration (if clinically feasible).

### MRI examination

1. Pre-MRI screening: Confirmation of pediatric fever (≥37.3°C) or critical status mandates cautious imaging protocol implementation.
2. Children may struggle to cooperate during MRI scan due to various factors. Infants under 3 months old can be managed through wrapping and feeding, avoiding sedation. For children over 3 months, non-pharmacological methods such as distraction and comforting techniques can be attempted first. If sedation is necessary, it can be administered orally or by enema 30 minutes before the scan. In cases of difficult cooperation, especially with children at risk of bleeding from nasal or sinus tumors, MRI can be conducted under general anesthesia to prevent image artifacts, injuries, and tumor bleeding (3)..
3. Wearing earplugs or noise-canceling headphones to protect hearing.
4. Choosing stable gadolinium contrast agents with low brain deposition rates. Administer the same dosage to children as to adults, but lower doses (0.05-0.08 mmol/kg) are recommended for premature infants and infants (4, 5). Following contrast agent injection, flush the catheter with a sufficient amount of normal saline.
5. Post-procedural care for MRI aligns with that established for CT.

### Pregnant and lactating women

#### CT examination

The administration of ionizing radiation during CT scanning necessitates rigorous risk-benefit analysis before imaging pregnant individuals. While the radiation dose in a single nasal and sinus CT scan is typically below the level considered harmful to fetal development (50–100 mGy) (6), guidelines advise against performing the scan during the initial trimester of pregnancy and suggest cautious use in the second and third trimesters. However, terminating a pregnancy solely due to exposure to diagnostic-level radiation is not recommended. Adequate shielding for the abdomen is essential during the procedure. For instance, when necessary to detect bone damage, abnormal bone formation, or tumor changes, low-dose CT scans can be cautiously employed with proper abdominal shielding and the briefest scanning duration possible. Non-contrast CT has minimal impact on breast tissue and lactation, allowing immediate breastfeeding. Contrast-enhanced CT historically advised cessation of breastfeeding and discarding milk for 24 hours. However, less than 1% of water-soluble iodine contrast is excreted into breast milk, with infants receiving only 0.01% of the maternal dose. The American College of Radiology (ACR) and American College of Obstetricians and Gynecologists (ACOG) recommend continuing breastfeeding, suggesting increased water intake post-exam to enhance contrast agent elimination (7).

#### MRI examination

MRI is preferred during pregnancy and lactation due to its non-ionizing properties. However, gadolinium-based contrast agents (GBCAs) should be used cautiously as they cross the placental barrier. The ACR advises avoiding MRI in the first trimester unless clinically urgent, citing theoretical risks of fetal malformations, thermal injury, and hearing impairment (8). GBCAs are not recommended for pregnant women unless essential for diagnosis or prognosis, as they release toxic gadolinium ions in fetal circulation (9). For lactating women, non-contrast MRI is safe. GBCAs appear in breast milk at <0.04% of the maternal dose within 24 hours, with <1% absorbed by infants. The ACR states breastfeeding need not be interrupted, though a 24-hour pause may be considered (10). Informed consent is required before contrast-enhanced MRI.

## Application of CT and MRI examinations in nasal cavity and paranasal sinus tumors

Preoperative imaging is used for tumor localization, assessing malignancy, and delineating lesion extent for staging. CT imaging effectively reveals the bony structures of the nasal cavity and paranasal sinuses, accurately detecting tumor-induced bone resorption or destruction and its extent. It holds particular diagnostic value for tumors originating from bone or cartilage. Benign tumors typically show bone resorption and remodeling, whereas malignant tumors often exhibit bone destruction. Squamous cell carcinoma (SCC) and adenoid cystic carcinoma (ACC) frequently involve perineural invasion, which CT evaluates through indirect signs such as bone resorption, destruction, and asymmetric widening of bony foramina (11). Ossifying fibroma presents with a variable-thickness bony shell at the tumor margin, alongside needle-shaped or patchy calcification and ossification within the tumor. In fibrous dysplasia, affected bones thicken and enlarge, displaying a homogeneously dense, ground-glass-like appearance. Both benign and malignant cartilaginous tumors on CT typically show clumped or scattered ossification. Additionally, CT also helps detect anatomical variations in the nasal and paranasal sinuses preoperatively. MRI offers superior soft-tissue resolution, enabling precise characterization of tumors. This includes delineating internal signal heterogeneity, boundaries, and depth of invasion into marrow and neurovascular structures (12). T2WI demonstrate clear differentiation between hypointense neoplasms and hyperintense inflammatory mucosa or protein secretions, a capability lacking in CT. In SCC and ACC, perineural invasion manifests as nerve signal replacement, thickening, and surrounding fat signal loss (13, 14). Skull base malignancies exhibit MRI-detectable alterations in the cavernous sinus and dura, including thickening (≥2mm) and nodular enhancement, as well as parenchymal infiltration (15). While CT excels at detecting subtle cortical erosion, MRI can identify marrow infiltration in the absence of cortical destruction. Advanced MRI techniques, such as diffusion-weighted imaging (DWI) and dynamic contrast-enhanced (DCE) MRI, facilitate differentiation between benign and malignant lesions, as well as subtype stratification of malignancies (16). Preoperative staging integration, using modified Kadish/Hyams criteria for olfactory neuroblastoma prognosis (17), and the Radkowski system for assessing sphenoid infiltration in juvenile nasopharyngeal angiofibroma (JNA), underscores the prognostic value of imaging, where the initial extent of the lesion directly correlates with clinical outcomes (18, 19).

CT is commonly used for intraoperative navigation to aid surgeons in real-time tumor localization, ensuring surgery precision and safety. CT angiography can identify feeding arteries and draining veins of tumors, such as in nasopharyngeal angiofibroma where the external carotid artery branches supply blood. It can also detect variations like kissing internal carotid arteries. Real-time MRI navigation enhances tumor resection accuracy, minimizes nerve and vessel damage, and optimizes normal tissue preservation compared to CT navigation.

Postoperative examinations focus on follow-up and efficacy evaluation. CT is crucial for assessing bone structure restoration, detecting recurrent lesions in areas like the skull base, orbit, and pterygopalatine fossa. They are particularly vital for postoperative monitoring of malignant bone and cartilage tumors or aggressive tumors like SCC. CT can identify various issues such as adhesions, fluid accumulation, soft tissue changes, lymph node enlargement, and surgical site alterations, indicating potential tumor recurrence. CE-CT can also evaluate abnormal blood supply, like tumor neovascularization. To minimize radiation exposure, low-dose CT is recommended for patients needing frequent monitoring. MRI can detect early signs of soft tissue repair, residual, or recurrent lesions. For instance, postoperative scars exhibit low signals in the T2WI sequence, whereas recurrent lesions manifest high signals or significant enhancement (20). Local recurrence may occur in 40-50% of JNA cases (21), Postoperative imaging indicating tumor residue and residual feeding arteries post-embolization increases the likelihood of recurrence (22). Sinonasal inverted papilloma (SNIP) presents a high recurrence rate, with 10% of cases potentially progressing to SCC (23), regular postoperative MRI follow-up is advantageous for early detection of malignant tissues. While distinguishing some recurrent tumors from postoperative granulation tissue on T1WI and T2WI is challenging, the apparent diffusion coefficient (ADC) value and DCE-MRI can be helpful (24–26). MRI Chemical Exchange Saturation Transfer (CEST) enhances tumor detection sensitivity and molecular-level image resolution. Additionally, MRI outperforms CT in discriminating radiation-induced osteonecrosis from intramedullary tumor recurrence. Differentiating bone marrow edema from intramedullary tumor infiltration is crucial during post-radiotherapy monitoring. Analysis of the time-signal enhancement curve in dynamic contrast-enhanced scans aids in distinguishing tumor activity from necrosis (14, 27). Follow-up assessments should also incorporate clinical physical examinations and nasal endoscopy. Vigilant monitoring is essential for high-risk recurrence and complex anatomical regions like the skull base, orbit, and pterygopalatine fossa. When necessary, both CT and MRI examinations should be conducted.

## Imaging diagnosis of nasal cavity and paranasal sinus tumors

The complex anatomical structure of the nasal cavity and paranasal sinuses-comprising bone, cartilage, mucosal epithelium, nerves, blood vessels, muscles, salivary glands, and lymphatic tissues, and bordered by the orbit, oral cavity, nasopharynx, and skull base-makes the origin of masses in these regions intricate. Imaging studies now recommend classifying tumors in the nasal cavity and paranasal sinuses into bone-derived and non-bone-derived categories. This paper provides a concise overview of both benign and malignant tumors in these areas, referencing the 2022 World Health Organization (WHO) pathological classification of nasal cavity and paranasal sinus tumors.

### Benign tumor

#### Sinonasal inverted papilloma

**Figure 1:**
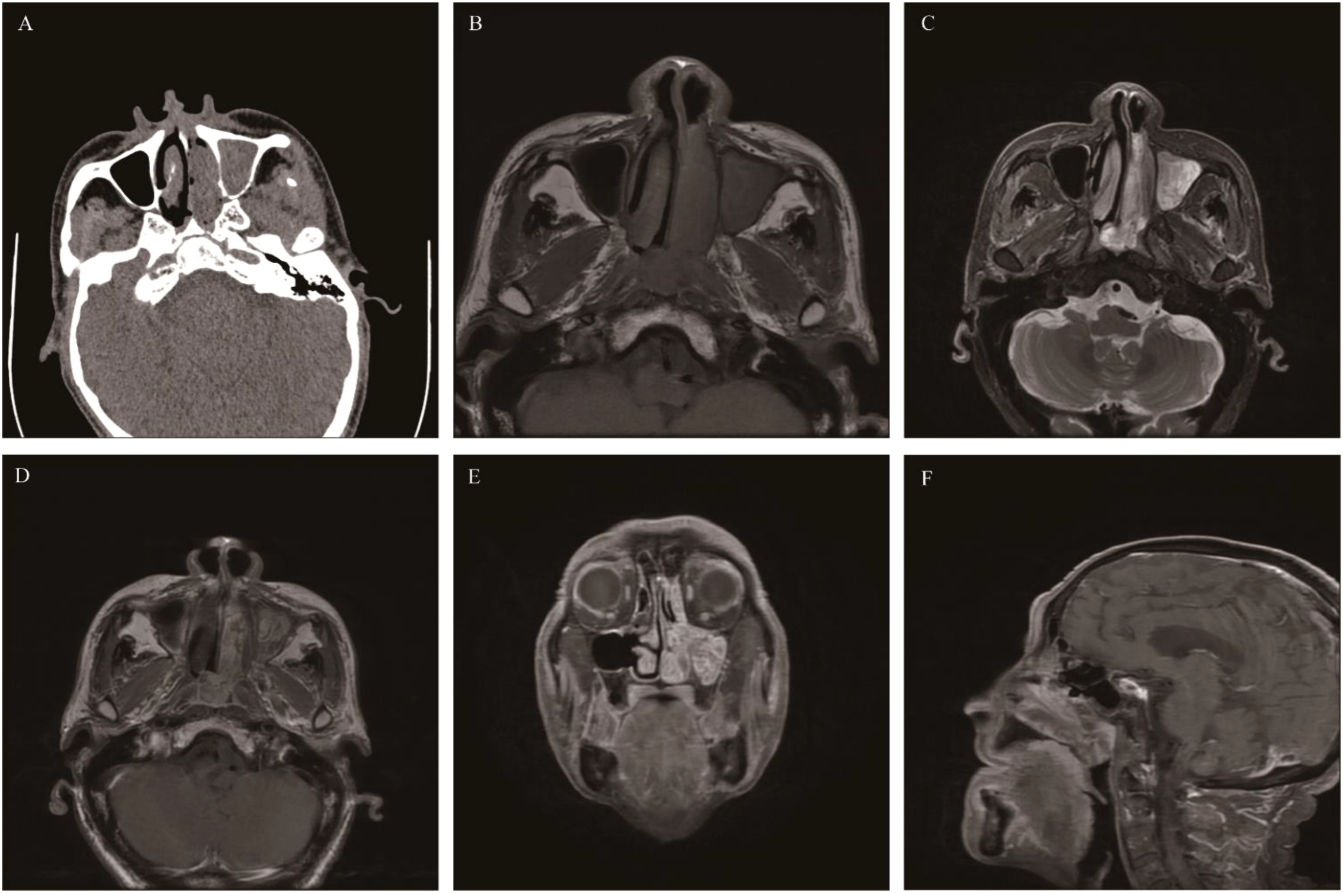
Sinonasal inverted papilloma: Axial CT scan showed a soft tissue mass in the left nasal cavity and maxillary sinus with mild bone resorption **(A)**. Axial T1WI **(B)** and Axial T2WI **(C)** showed the mass with slightly hypointense signal and heterogeneously slightly hyperintense signal respectively, Contrast-enhanced MRI displayed typical “CCP” in axial, coronal, and sagittal planes **(D-F)**.

A common benign tumor of sinus epithelial origin in the nasal cavity and paranasal sinuses, but it has a tendency for invasive growth, easy recurrence after surgery, and carcinogenesis (28). The onset of SNIP may be associated with HPV infection (29). The most common site of SNIP is the lateral wall of the unilateral nasal cavity near the middle meatus, and it spreads and fills the ipsilateral nasal cavity and paranasal sinuses, with the maxillary sinus being the most frequently affected. SNIP mainly affects men aged 40–70 years (30). The clinical manifestations lack specificity, and the common symptoms are mainly unilateral persistent and progressive nasal obstruction and anterior and/or posterior rhinorrhea. CT: Soft-tissue density, higher than that of the nasal concha and similar to muscle. Microcalcifications are present in 20% of cases (31). Large-sized SNIP causes compression and displacement of the middle and inferior nasal conchae and expansive changes of the maxillary sinus ostium bone with bone resorption. In some cases, a “bubble sign” can be seen during the process of the tumor filling the nasal cavity and paranasal sinuses, which represents the residual gas between the lesion and the intrinsic structures of the nasal cavity and paranasal sinuses. In some cases, bone hyperplasia and sclerosis occur at the base of the tumor, which is one of the signs suggesting SNIP. The reason is that the long-term inflammatory reaction at the origin site of SNIP stimulates osteogenesis. MRI: Iso-or slightly low signal on T1WI, heterogeneous slightly high signal on T2WI. The enhanced signal on T2WI and T1WI shows a striated structure with alternating high and low signals, often described as “convoluted cerebriform pattern, CCP”. CCP is one of the relatively characteristic imaging findings of SNIP. SNIP easily obstructs the ostiomeatal complex, thus leading to a large amount of mucus retention in the paranasal sinuses and obstructive sinusitis. MRI can easily distinguish the boundary between the tumor and inflammation. When necrosis occurs inside SNIP, the CCP signal is absent, and there is invasion of structures outside the nasal cavity and paranasal sinuses, the possibility of SNIP malignancy should be alerted (32) (**Figure 1**).

#### Juvenile nasopharyngeal angiofibroma

A benign but invasive vascular tumor, accounting for 0.5% of all head and neck tumors (33). It mainly occurs in the sphenopalatine foramen or posterior nasal cavity. JNA is predominantly seen in adolescents and young males, rarely in females, with an average age of 17 years (34). The most common symptoms are unilateral nasal obstruction and recurrent epistaxis. It is prone to local invasion of the intracranial and orbital regions, which can cause headache and diplopia (35).CT: It is located in the sphenopalatine foramen area or centered in the pterygopalatine fossa, presenting as an expansively growing soft-tissue mass with a density similar to that of muscle. The adjacent sphenoid sinus and pterygoid process often show compression, reshaping, bone destruction, and enlargement of the sphenopalatine foramen. Recently, some scholars have proposed the Holman-Miller sign (36), which refers to the anterior bowing of the posterior wall of the maxillary sinus, resulting from bone remodeling and compression of the posterior wall of the maxillary sinus due to JNA growth. Since JNA is composed of blood vessels and a fibrocyte-rich extracellular matrix, it shows obvious and uniform enhancement on contrast-enhanced CT, with an enhancement degree almost equivalent to that of blood vessels. MRI: It shows isointense or hypointense signals on T1WI, similar to or slightly lower than the muscle signals. The signals on T2WI are heterogeneous, mainly because the vascular matrix shows hyperintense signals, while the fibrous components and vascular flow void effects show hypointense signals. Vascular flow void signals are seen inside and around JNA, which together with the hyperintense signals of the tumor parenchyma present the “salt-and-pepper sign”. The enhancement degree of JNA is significantly higher than that of the surrounding tissues. In addition, research reports that the ADC value of JNA is higher compared with other benign tumors (37).

#### Schwannoma

**Figure 2:**
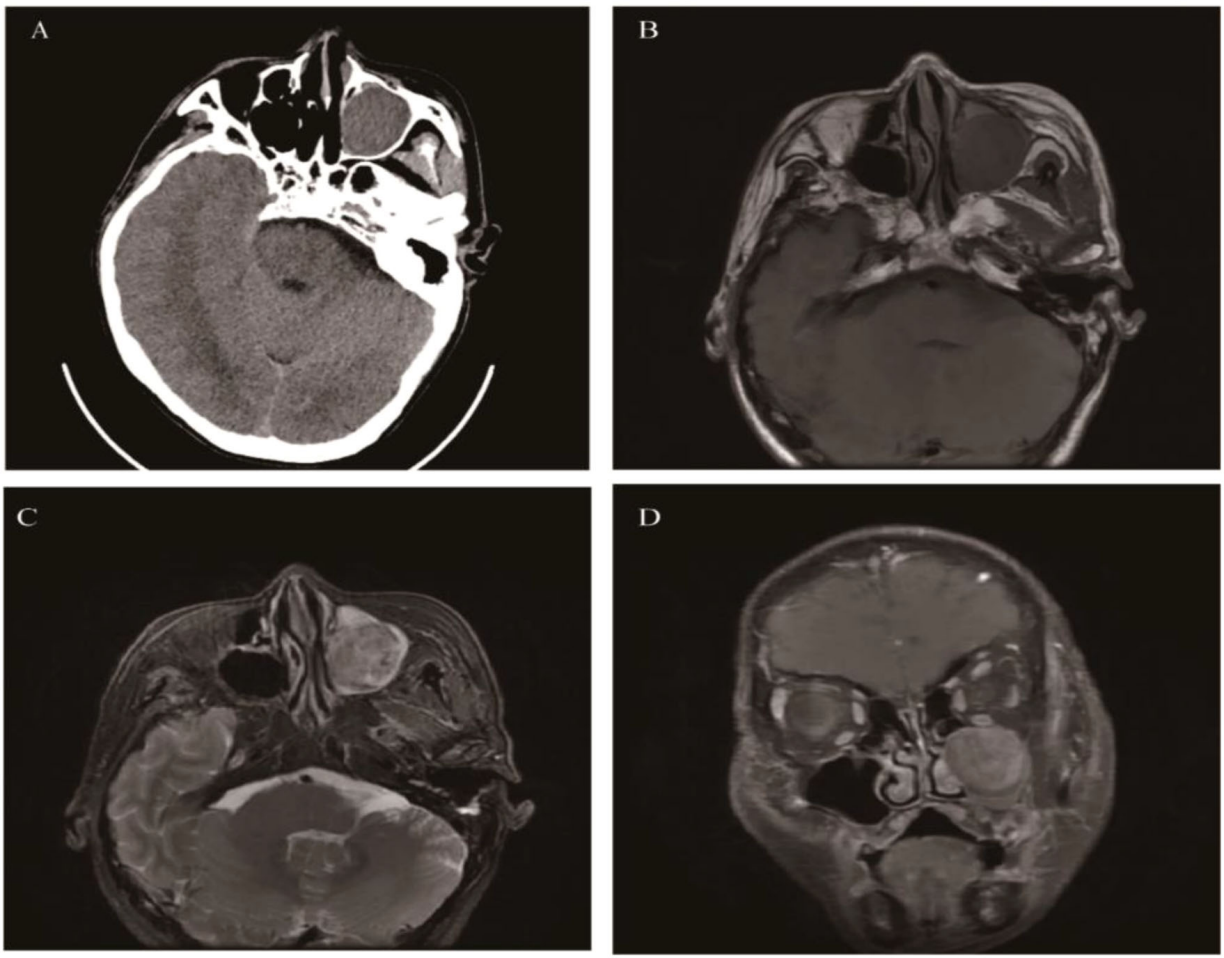
Schwannoma: Axial CT showed a well-defined, round soft tissue mass in the left maxillary sinus causing slight sinus cavity enlargement **(A)**. Axial T1WI **(B)** and Axial T2WI **(C)** exhibited isointense and heterogeneous hyperintense signal intensity tissue which showed moderate enhancement on coronal contrast-enhanced images **(D)**.

A predominantly benign tumor originating from Schwann cells of peripheral nerves. Schwannomas occurring in the nasal cavity and paranasal sinuses account for 4% of those in the head and neck region (38). Most of them originate from the ophthalmic and maxillary branches of the trigeminal nerve or Schwann cells of the autonomic nerves, so they mainly occur in the sphenoid sinus, nasal septum, and the anterior part of the lateral nasal wall, followed by the maxillary sinus (39–41). Some schwannomas may be accompanied by intracranial extension. This disease is more common in middle-aged people aged 30–60 years, with no significant gender difference. The most common form is a unilateral tumor in the nasal cavity or paranasal sinuses, but bilateral tumors should raise a high suspicion of neurofibromatosis. The clinical manifestations are not characteristic. Larger tumors mainly present with nasal obstruction, decreased sense of smell, facial pain and numbness, and abnormalities in the eyeball and vision. CT shows a round-like, well-defined soft-tissue mass in one nasal cavity or paranasal sinus, with uniform density, and uneven density due to cystic degeneration and necrosis. Some tumors have punctate or strip-like calcifications. The tumor grows slowly. The bone in contact with the tumor shows bone resorption, thinning, and mild displacement, but bone destruction is rare. CT enhancement shows moderate to marked homogeneous enhancement, and the cystic area shows no enhancement. MRI: isointense or slightly hypointense on T1WI. T2WI shows heterogeneous hyperintensity, and the signal of the solid part is similar to that of the brainstem (41). Pathologically, schwannomas have an Antoni A area composed of solid parts and an Antoni B area composed of cystic parts. When the Antoni A area is located in the center of the lesion and the Antoni B area is located at the edge of the lesion, a characteristic “target sign” can be formed on T2WI, that is, slightly hypointense in the center of the lesion and hyperintense at the edge. The degree of MRI enhancement is the same as that of CT enhancement (**Figure 2**).

#### Ectopic pituitary adenomas

**Figure 3:**
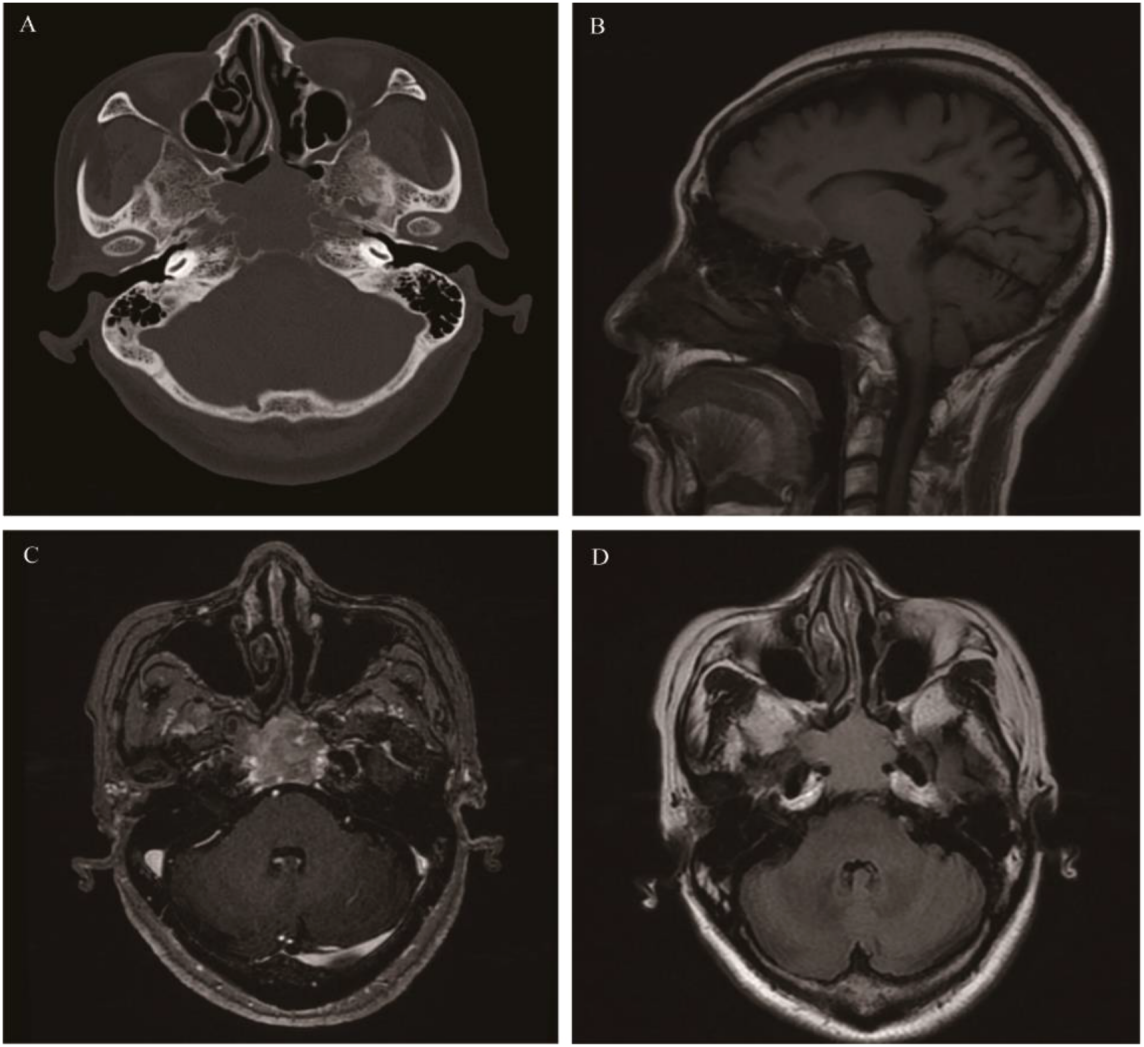
Ectopic pituitary adenomas: Axial CT showed an irregular soft tissue density mass involving the sphenoid sinus and clivus, with bone destruction mainly characterized by bone resorption and bone sclerosis formation **(A)**. It appeared as isointense on T1WI **(B)**, slightly hyperintense on T2 Fluid Attenuated Inversion Recovery (T2 FLAIR) **(C)**, and showed significant enhancement on MR contrast-enhanced scan **(D)**.

EPAs are extremely rare tumors, and most of the statistical data are derived from case reports. EPAs are located outside the Sella turcica and originate from the anterior pituitary gland tissue. They are more common in middle-aged and elderly people aged 40-70, with no significant gender difference. Approximately 60% of the lesions are located in the sphenoid sinus and suprasellar region, followed by the clivus, nasal cavity, and cavernous sinus (42). This may be related to the migration path of the Rathke’s pouch during embryonic development (43). The clinical manifestations depend on the mass effect, invasive behavior, and hormone levels of the tumor. EPAs located in the cavernous sinus and clivus compress the cranial nerves, leading to visual impairment and facial paralysis (44). EPAs occurring in the sphenoid sinus can cause cerebrospinal fluid rhinorrhea (45). Since most tumors (75%) are functional, they can lead to Cushing’s syndrome, acromegaly, and hyperthyroidism (46). CT: The tumor appears as an isodense or slightly hyperdense mass. Expansive changes in the sphenoid sinus, bone resorption, or remodeling of the sphenoid sinus wall are common, but bone destruction is relatively rare. There is moderate to marked enhancement after contrast-enhanced CT. MRI: The tumor shows isointensity on T1WI, slightly hyperintensity on T2WI, and mild to moderate enhancement. Signal heterogeneity is caused by hemorrhage and cystic degeneration (**Figure 3**).

#### Giant cell tumor of bone

**Figure 4:**
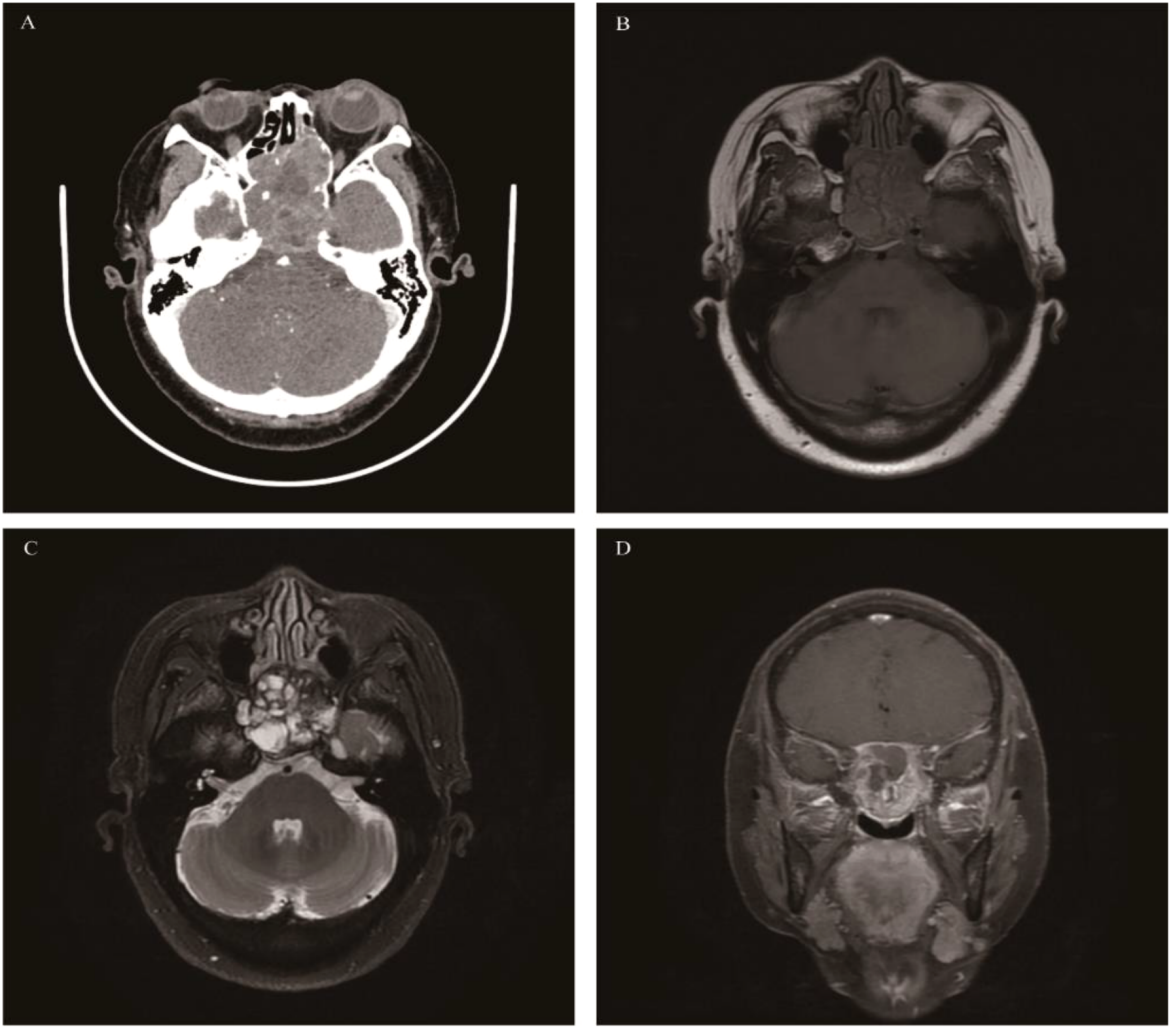
Giant cell tumor of bone: Axial CT enhancement revealed an irregular, hyperintense mass causing extensive bone damage in the pterygoid sinus, slope, and posterior sieve sinus **(A)**. The mass displayed the typical “soap bubble-like” appearance on T1WI and T2WI, akin to GCTB found in the metaphysis of long bones **(B, C)**, with the solid part of the mass demonstrating significant enhancement on coronal fat-saturated contrast-enhanced T1WI, while the liquid component does not **(D)**.

A benign yet locally invasive tumor composed of osteoclast-like giant cells and osteoclast precursor cells. Less than 2% of GCTB occur in the head and neck. In cases involving the nasal cavity and paranasal sinuses, the lesions mainly occur in the sphenoid sinus (47, 48), followed by the maxillary sinus, and rarely in the nasal cavity. The affected population is mostly between 20 and 45 years old, with a median age of 25 years. The incidence in females is slightly higher than that in males (49). Since GCTB in the nasal cavity and paranasal sinuses mainly occurs in the sphenoid sinus, the clinical symptoms are mainly visual impairment, visual field defect, diplopia, hyposmia, and pituitary dysfunction. On CT, GCTB appears as a soft-tissue density mass with expansive bone destruction, but most of the bone cortex is intact, and periosteal reaction is basically invisible. It shows low or isointense signals on T2WI, and the low signal on the T2WI sequence is of great significance for the diagnosis of GCTB. T1WI shows a heterogeneous signal mainly with low signal. Of course, the “soap bubble” sign is also one of the important signs for the diagnosis of GCTB. The obvious circular T2WI low signal intensity around the tumor is an important feature of atypical GCTB, which is consistent with the feature of circular T2 low signal around the tumor of GCTB occurring in long bones reported in the literature (50) (**Figure 4**).

### Malignant tumors

#### Squamous cell carcinoma

**Figure 5:**
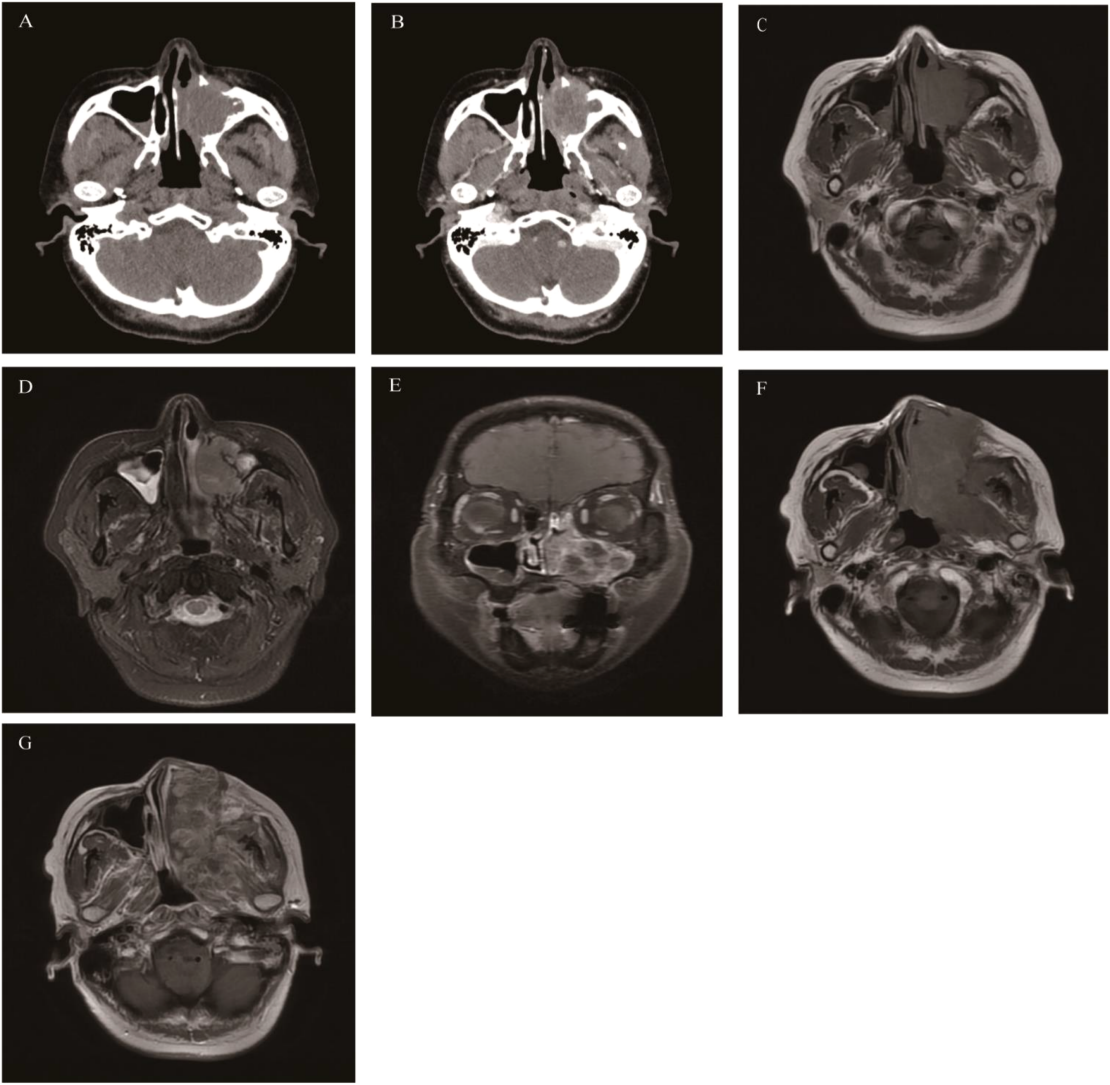
Squamous cell carcinoma: Axial CT showed an irregular soft tissue mass with bone destruction in the left maxillary sinus, exhibiting moderate inhomogeneous enhancement **(A, B)**. T1WI showed an isointensity while T2WI indicated a slightly hyperintensity, both with moderate heterogeneous enhancement **(C-E)**. Nine months later, the mass had significantly enlarged, extending anteriorly to the facial skin and posteriorly to the left parapharyngeal space on **(F, G)**.

The most common malignant tumor of the nasal cavity and paranasal sinus mucosal epithelium, accounting for 80% of nasal cavity and paranasal sinus malignancies (51). It is more common in men over 50 years old. The etiological factors for tumorigenesis include: smoking, chronic inflammation, nasal polyps, irritation from nickel, arsenic, chromium, leather, dust, isopropyl alcohol, etc., low immunity, malignant transformation of inverted papilloma, radiotherapy, and HPV infection (52). Moreover, the latest literature supports that HPV-positive SCC has a better prognosis than HPV-negative SCC (53–55). SCC occurs in the maxillary sinus (60%) and nasal cavity (25%), and less frequently in the ethmoid sinus, frontal sinus, and sphenoid sinus (56). CT: A unilateral irregular soft- tissue mass. Generally, the tumor is relatively large in size, with non-uniform density, unclear boundaries, disappearance of the maxillofacial fat space, involvement of adjacent sinuses and nasal cavity, and obvious bone destruction, resulting in the disappearance of the normal contour of the nasal cavity and paranasal sinuses. MRI: The SCC tumor itself shows low or equal signal on T1WI and slightly high signal on T2WI. Cystic changes and hemorrhage are commonly seen inside larger SCCs, and the signals of cystic changes are often located in the center of the tumor. MRI enhancement: Small-sized SCCs show uniform enhancement, while larger lesions show non-uniform enhancement, mostly moderate to severe non-uniform enhancement. The ADC value is usually higher than that of lymphoma but lower than that of ACC (57). Regional lymph node metastasis of SCC in the nasal cavity and paranasal sinuses is very common (**Figure 5**).

#### Adenoid cystic carcinoma

A malignant tumor originating from salivary gland epithelium, it is the most common malignant tumor of minor salivary glands in the head and neck. ACC occurring in the nasal cavity and paranasal sinuses is generally considered to originate from ectopic minor salivary glands here, accounting for 10%-20% of head-and-neck ACC. In statistical cases of nasal cavity and paranasal sinus ACC, the maxillary sinus is the most commonly affected site, followed by the nasal cavity, ethmoid sinus, and sphenoid sinus. The disease occurs between the ages of 5 and 60, with a slight female predominance (58–60). The tumor grows slowly with insidious symptoms. Regional lymph node metastasis is rare, but hematogenous metastasis mainly to the lungs is common. ACC appears as a relatively large mass on CT or MRI. CT: It presents as a large, “ginger-shaped” soft-tissue mass. In the pathological classification, ACC of cribriform and tubular types shows cystic areas of varying sizes, while the solid-type ACC with the highest malignancy grade does not show cystic areas. The bone destruction of the paranasal sinuses by ACC often shows both expansile and erosive features, and the degree of bone destruction by ACC is lower than that by SCC. Nerve invasion is a characteristic manifestation of ACC. On CT, the enlargement and bone destruction of the pterygopalatine fossa and its adjacent foramina such as the foramen rotundum, foramen ovale, and foramen spinosum highly suggest ACC.

**Figure 6:**
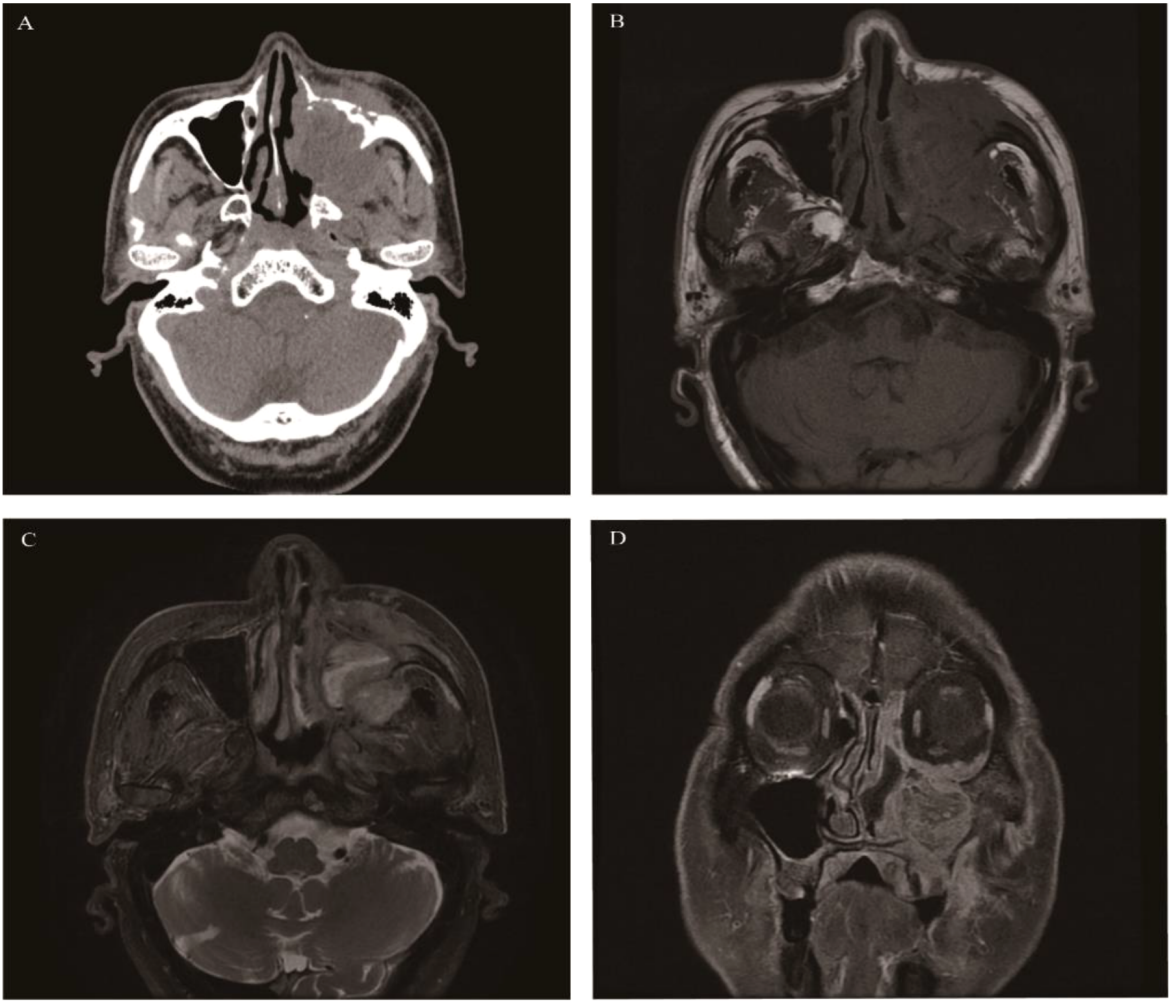
Adenoid cystic carcinoma: Axial CT revealed an irregular soft tissue mass in the left maxillary sinus with notable but less extensive bone destruction compared to SCC **(A)**. On T1WI, the mass appeared isointense **(B)**, while on T2WI **(C)**, it exhibited a slightly hyperintense signal with strip- or tube-shaped hyperintense regions. Coronal contrast-enhanced T1WI displayed a heterogeneous “sieve-sac-like” pattern **(D)**. Additionally, enhanced MRI indicated infiltration of the mass into the orbit, hard palate, and buccal mucosa.

MRI: The signal on T1WI and T2WI is mainly isointense. The cribriform and tubular types of ACC show dense and uniformly-sized cystic areas. Due to different cystic components, T1WI may show high or low signal. The solid-type ACC shows no cystic areas, and the signal on T2WI is significantly reduced. Typical ACC show relatively low signal on DWI, and the ADC value is higher than that of other pathological types of malignant tumors. The nerve invasion of ACC on MRI is manifested as nerve thickening, enhancement, disappearance of normal nerve structure accompanied by a soft-tissue mass, disappearance of perineural fat signal, and atrophy of the muscles innervated by the nerve, etc. (14) (**Figure 6**).

#### Sinonasal undifferentiated carcinoma

**Figure 7:**
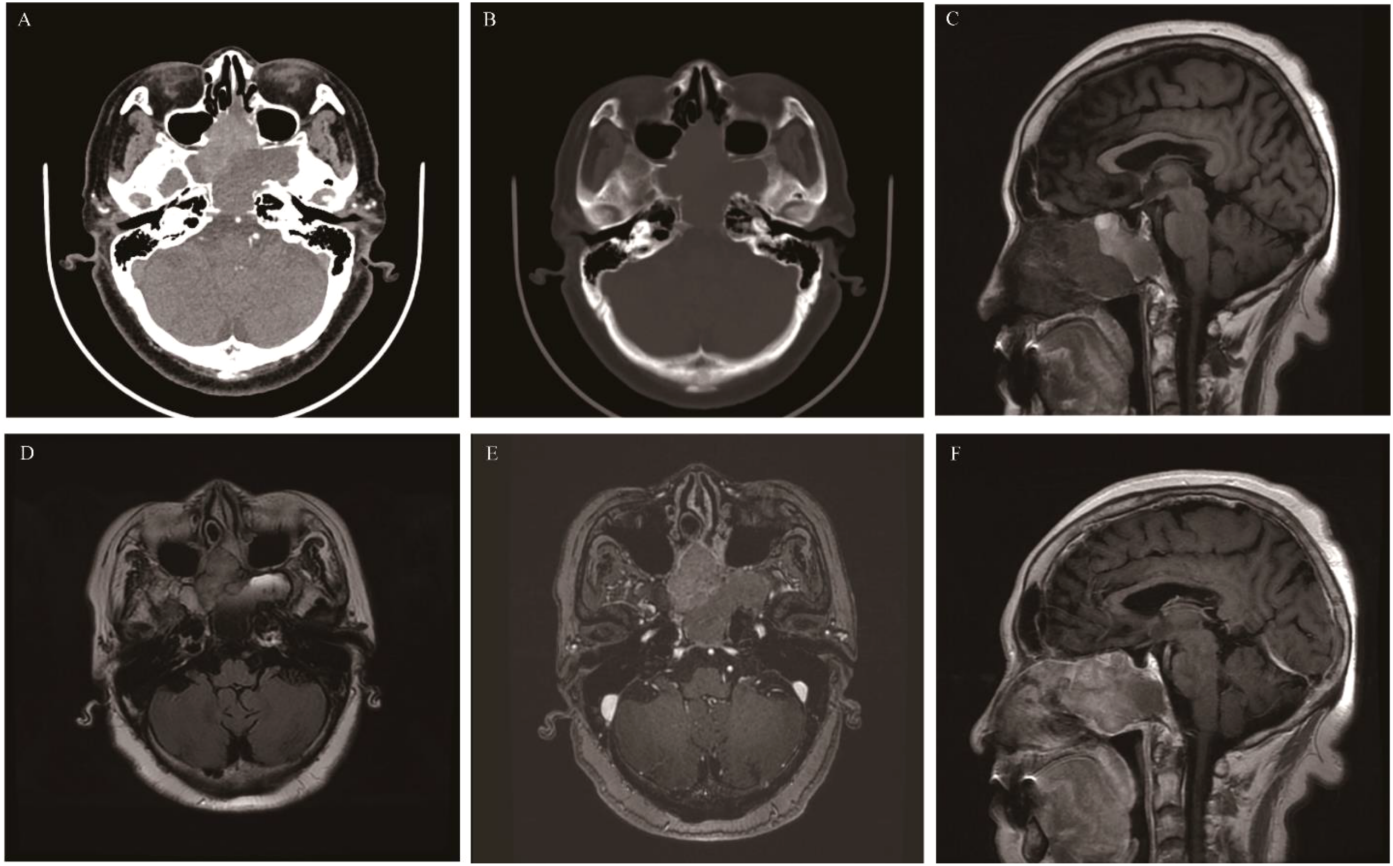
Sinonasal undifferentiated carcinoma: Axial CT showed the mass was mainly located at the top of the nasal cavity and involved the sphenoid sinus, ethmoid sinus, and clivus **(A)**. The mass caused obvious bone destruction **(B)**. It presented isointense on T1WI and isointense on T2WI, with heterogeneous enhancement on contrast-enhanced MRI On axial and sagittal **(C-F)**.

In 2022, the WHO defined SNUC as a high-grade epithelial tumor without any lineage differentiation (61). Currently, most literature supports that SNUC is a surface (Schneiderian) epithelial-derived malignancy (62). SNUC can occur across a wide age range (20–90 years), with a median age of 50–60 years, and it is slightly more prevalent in males than females. Nasal obstruction and epistaxis are common clinical symptoms. Proptosis, visual impairment, and cranial nerve palsy may be observed as the disease progresses (63). SNUC is difficult to cure completely and has a very poor prognosis. Recurrence, lymph node metastasis, and distant metastasis are more common than in other malignancies. Literature reports that approximately 10%-30% of SNUC patients have lymph node metastasis at the time of diagnosis (64). SNUC typically arises in the ethmoid sinuses and the roof of the nasal cavity, often involving multiple sinuses. It is difficult to determine the origin of large tumors, and they can easily cause bone destruction and involve the orbit, pterygopalatine fossa, cavernous sinus, parapharyngeal space, and the dura mater of the anterior/middle cranial fossa (65). On CT, it appears as an irregular soft tissue mass with a blurred border and uniform density, but shows heterogeneous enhancement after contrast administration. MR signals are not significantly specific (**Figure 7**). Necrosis may occur in large tumors (>4 cm), with marked heterogeneous enhancement. The diagnosis of SNUC relies on pathology and requires the exclusion of other malignancies in the nasal cavity and paranasal sinuses. CT and MRI are mainly used for tumor localization and to determine the extent of the lesion for Tumor Node Metastasis (TNM) or Kadish staging (66).

#### Olfactory neuroblastoma

**Figure 8:**
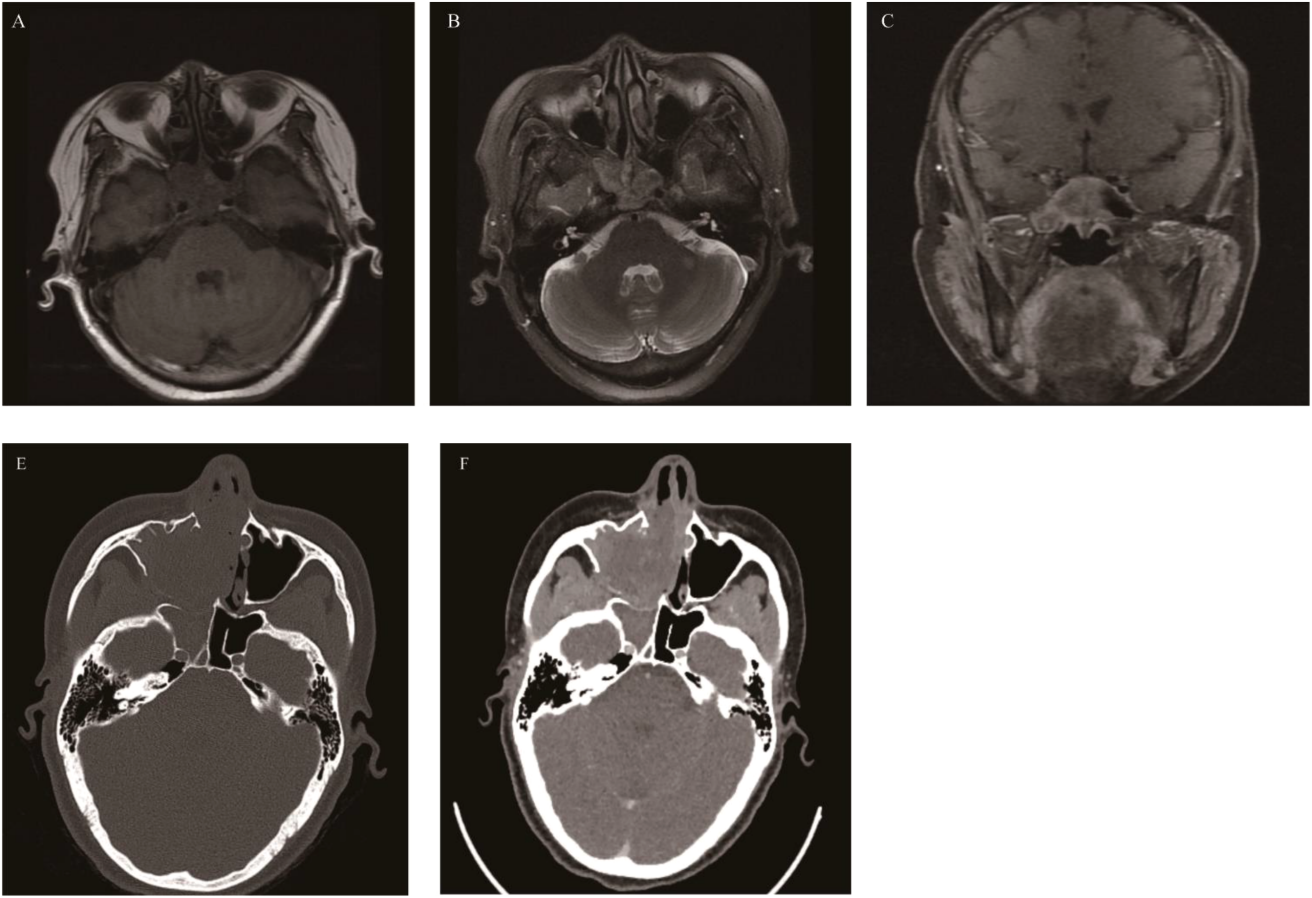
Olfactory neuroblastoma: Patient 1: An irregular mass was found in the sphenoid sinus, presenting as hypointense on T1WI, hyperintense on T2WI, and showing moderate enhancement on MRI contrast **(A-C)**. Patient 2: Axial CT showed a soft-tissue density mass in the right maxillary sinus and sphenoid sinus with obvious bone destruction, and heterogeneous enhancement on CT contrast **(E, F)**. The locations of the masses in both patients were atypical.

Malignant tumors originating from the neural crest of the olfactory nerve epithelium are generally considered to be distributed in the olfactory region. Therefore, the tumors usually have a specific site of onset-the top of the nasal cavity or the lateral wall near the middle turbinate. The incidence accounts for approximately 3%-5% of malignant tumors in the nasal cavity and paranasal sinuses, with a slightly higher prevalence in males than females. According to the 5th edition of WHO, the age of onset ranges from 2 to 90 years, with a peak at 50–60 years (67). Recent studies have found that ONB in patients under 40 years old is not uncommon, and the grade is often higher (68).In the early stage, ONB is confined to the top of the nasal cavity. In the advanced stage, the tumor often involves multiple paranasal sinuses, the orbit, the optic nerve, and the frontal lobe brain parenchyma. The most common clinical symptom is nasal congestion. Other common symptoms include recurrent epistaxis and loss of smell. In the advanced stage, ONB may present with epiphora, diplopia, exophthalmos, and decreased visual acuity. When the frontal lobe is involved, headache and epilepsy may occur. Unilateral symptoms are more obvious than bilateral ones. CT findings show a “dumbbell-shaped” or “mushroom-shaped” mass located at the top of the nasal cavity and the anterior cranial fossa. When the mass is small, it has uniform density, similar to that of muscle. When the tumor is large, there are patchy necrosis and calcification. Most ONBs cause bone destruction in the ethmoid sinus, the orbital roof, and the frontoethmoidal complex. After enhancement, the solid part of ONB shows moderate to marked enhancement, and liquefactive necrosis leads to uneven enhancement of the tumor. MRI: Compared with muscle signals, ONB shows isointensity on T1WI and mainly slightly high-intensity on T2WI. Necrosis and calcification inside large tumors result in uneven tumor signals. The degree of enhancement on MRI is similar to that on CT. The latest research suggests that cystic changes at the edge of intracranial-involved tumors are indicative of ONB (69, 70) (**Figure 8**).

#### Malignant sinonasal melanoma

Rarely, it is a highly invasive malignant tumor. Its incidence in the nasal cavity and paranasal sinuses is approximately 4% (71, 72). The nasal cavity and paranasal sinuses are the predilection sites for MSM (accounting for about 80%), with the nasal cavity being more commonly affected than the paranasal sinuses. In the nasal cavity, the anterior lower part of the nasal septum is a common site, and in the paranasal sinuses, the maxillary sinus is frequently involved (73, 74). MSM often occurs unilaterally in the nasal cavity and paranasal sinuses, so the common clinical manifestations are unilateral nasal obstruction and epistaxis (75). Essentially, most of these tumors contain melanin, but 10%-30% of the lesions are hypopigmented or amelanotic melanomas. The population distribution is mainly among those aged 50–80 years, with no significant gender difference. Due to the rich blood supply and lymphatic drainage in the head and neck region, MSM is highly invasive, has a high recurrence rate, and is prone to multiple recurrences. In the advanced stage, it is likely to metastasize to the lungs, brain, bones, and liver. The literature reports that 10%-20% of MSM cases in the nasal cavity and paranasal sinuses present with cervical lymph node metastasis at the time of onset (76).CT findings: MSM shows an isodense appearance. Larger MSMs can cause compression of the adjacent paranasal sinus accompanied by varying degrees of bone destruction.

MRI findings: Referring to the muscle signal, typical pigmented MSM appears hyperintense on T1WI and hypointense on T2WI. Hypopigmented or amelanotic melanomas show low, isointense, or slightly hyperintense signals on T1WI and slightly hyperintense or hyperintense signals on T2WI. MSM has a relatively high tendency to bleed (approximately 50%), but there is usually hemosiderin deposition around the bleeding area, which can be distinguished from melanin. Due to the relatively rich blood supply of MSM, it shows obvious enhancement on both CT and MRI. The contrast-enhanced scan sequence can also provide a reference for distinguishing melanin, high-protein secretions, and bleeding lesions. The literature reports that the ADC value of MSM is generally lower than that of SCC (77).

**Figure 9:**
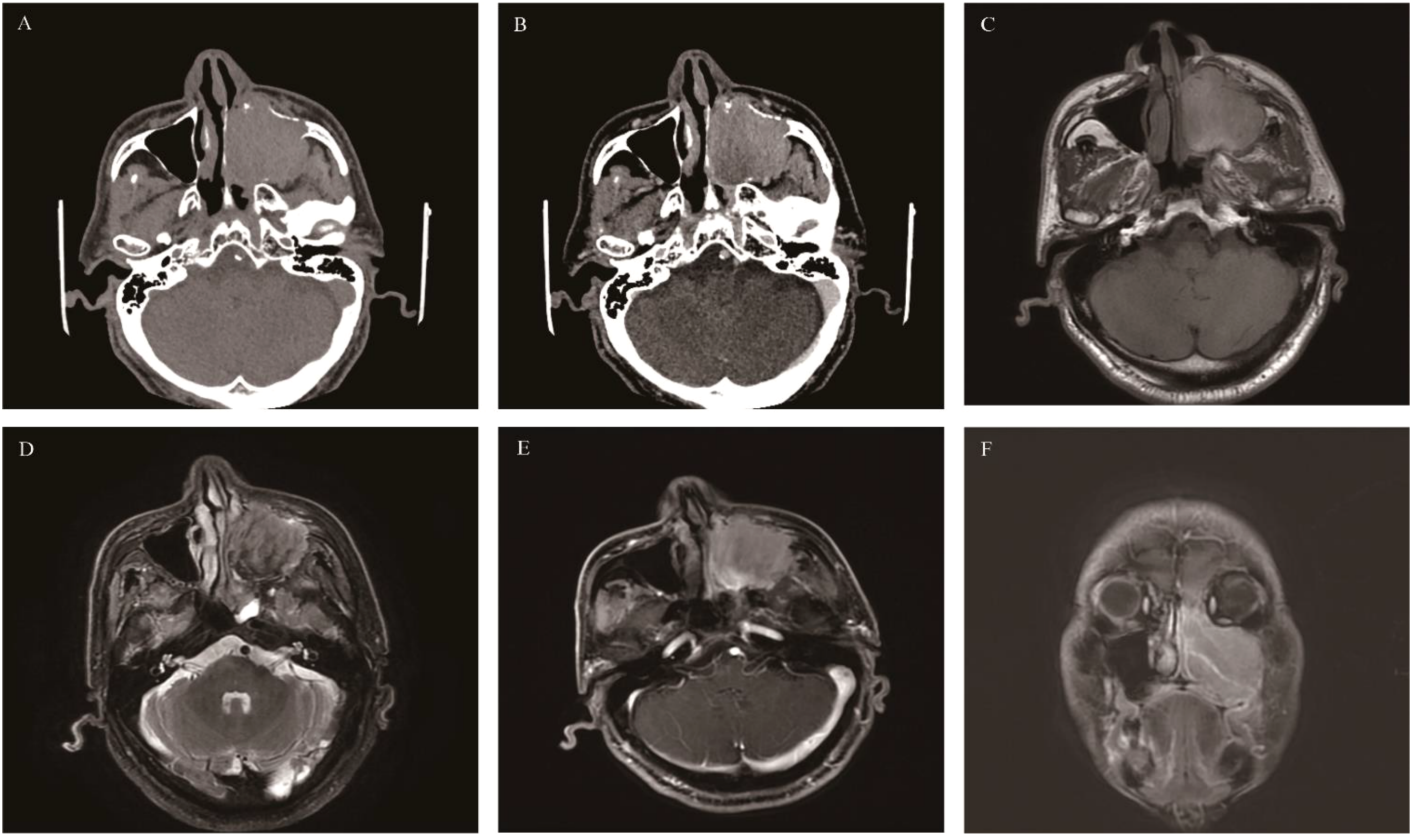
Malignant sinonasal melanoma: An irregular soft-tissue density mass in the left maxillary sinus was accompanied by obvious bone destruction, showing heterogeneous enhancement on CT **(A, B)**; On T1WI, the mass presented isointense signals with linear slightly hyperintense signals within it**(C)**. On T2WI, the slightly hyperintense mass contained multiple linear hypointense areas **(D)**. The mass showed obvious heterogeneous enhancement on axial and coronal MRI **(E, F)**.

“A septate pattern” (a sign of alternating high and low signals on non-contrast T1WI) may also highly suggest MSM (78) (**Figure 9**).

#### Rhabdomyosarcoma

**Figure 10:**
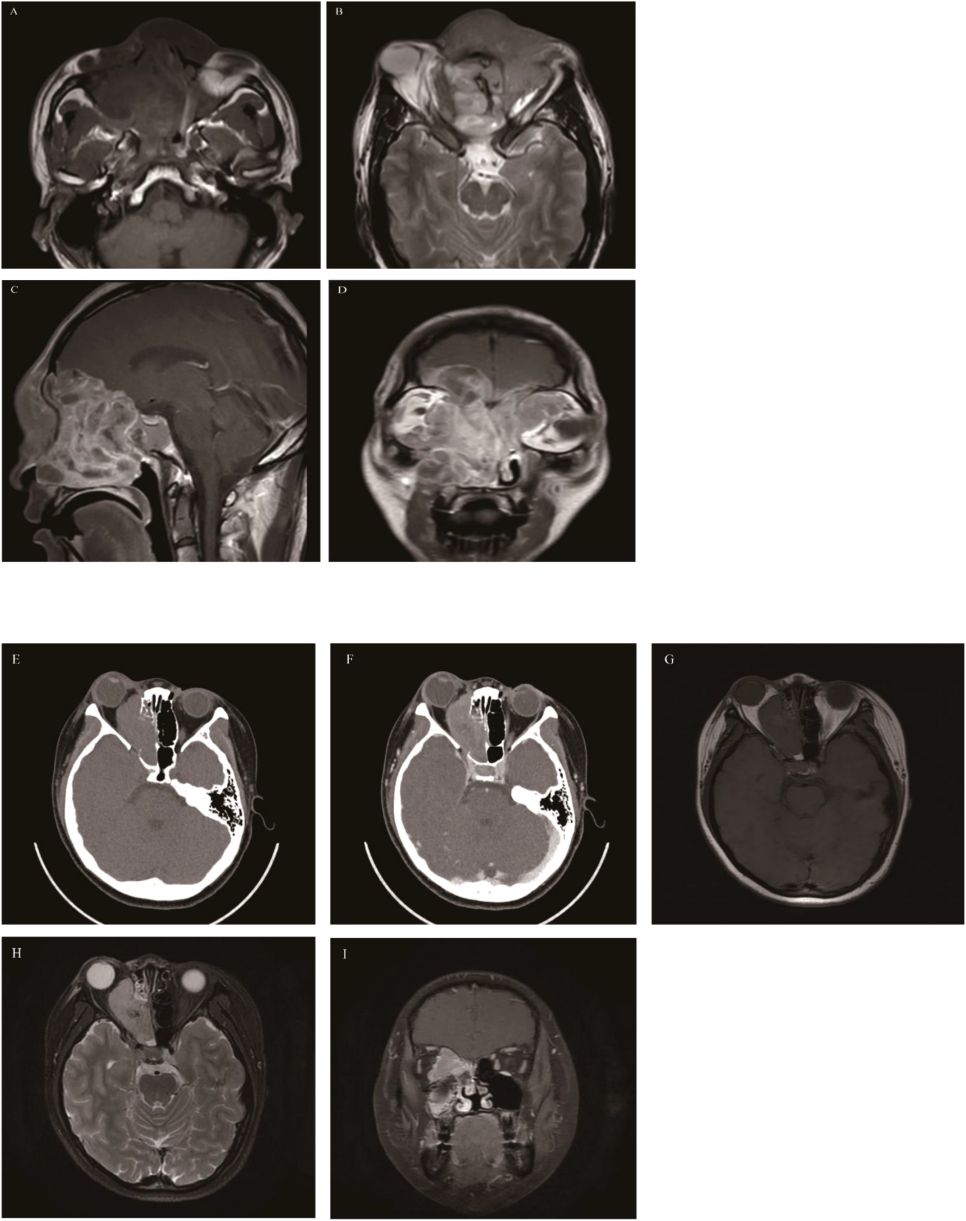
Embryonal rhabdomyosarcoma **(A-D)**: The mass involved the entire group of paranasal sinuses, nasal cavity, orbit, and intracranial region; The mass showed heterogeneous signal intensity, is isointense on T1WI **(A)**, hyperintense on T2WI **(B)**, and exhibited marked heterogeneous enhancement on MRI contrast enhancement **(C, D)**. Alveolar rhabdomyosarcoma **(E-I)**: On Axial CT, the mass was located in the right ethmoid sinus with homogeneous density, involved the right orbit with obvious bone destruction, and showed marked enhancement **(E, F)**. The mass appears isointense on T1WI **(G)**, hyperintense on T2WI **(H)**, and showed marked homogeneous enhancement on contrast-enhanced MRI **(I)**.

RMS is the most common malignant tumor in children and adolescents, mostly occurring in individuals under 20 years of age, and more prevalent in males than females (16, 79, 80). RMS originates from primitive mesenchymal cells that differentiate into skeletal muscle. 35%-40% of rhabdomyosarcomas occur in the head and neck region, and approximately 10-15% of head and neck rhabdomyosarcomas occur in the nasal cavity (81). Embryonal RMS (ERMS) is the most common subtype, followed by alveolar RMS (ARMS) (82), and the prognosis of ERMS is better than that of alveolar RMS.RMS is highly malignant and prone to invade adjacent tissues. Hematogenous metastasis is the main mode of metastasis for ERMS, which easily involves the bone marrow, lungs, cerebrospinal fluid, and abdominal organs, while lymph node metastasis is more common in ARMS.CT findings show a homogeneous soft-tissue density mass with obvious adjacent bone destruction. Larger tumors may have low-density liquefactive necrosis areas, and calcification is rare. ERMS is more likely to present with necrosis or mucoid degeneration than ARMS. After contrast-enhanced CT, the tumor shows moderate to marked heterogeneous enhancement, with a higher enhancement degree than that of muscle. MRI findings: On T1WI, the tumor shows isointense or slightly hypointense signals, and the hemorrhage within the tumor appears as hyperintense signals on T1WI. On T2WI, it shows heterogeneous hyperintense signals (presumably due to intratumoral hemorrhage, necrosis, and residual bone), but the overall signal is slightly higher than that of muscle. The enhancement pattern after contrast-enhanced MRI is similar to that of contrast-enhanced CT. Typical ERMS shows linear, circular, or grape-cluster-like enhancement, while ARMS mostly shows relatively homogeneous enhancement of solid components, and ARMS is more likely to invade the skull than ERMS (83) (**Figure 10**). Literature reports indicate that ADC of RMS in the nasal cavity and paranasal sinuses is 0.992 ± 0.133×10–3 mm2/s, which is significantly lower than that of paranasal sinus carcinomas (84).

#### Sinonasal teratocarcinosarcoma

**Figure 11:**
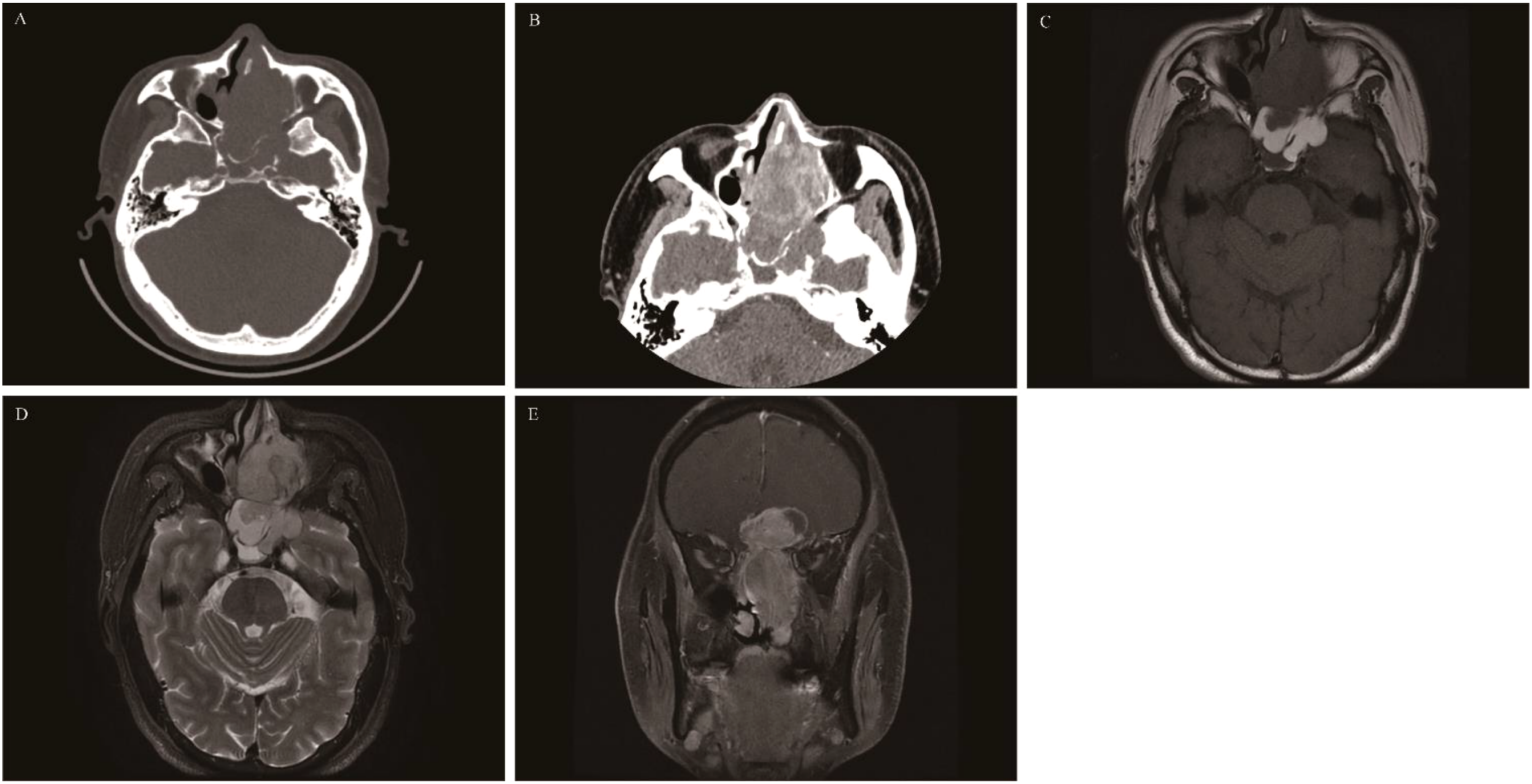
Sinonasal teratocarcinosarcoma: CT revealed an irregular soft tissue mass in the left ethmoid sinus with significant bone destruction and heterogeneous enhancement **(A, B)**. On T1WI the mass appeared isointense **(C)**, while on T2WI, it is slightly hyperintense **(D)**. The coronal contrast-enhanced image depicted a “dumbbell-shaped” mass with pronounced enhancement **(E)**, this presentation is often misdiagnosed as olfactory neuroblastoma.

A rare aggressive tumor. Currently, the literature reporting this tumor mainly consists of case reports or small-sample studies. The SNTCS tumor has a complex composition, predominantly containing neuroepithelial components, mixed with stromal components and a small amount of epithelial components. It mostly occurs in the nasal cavity, followed by the ethmoid sinus and maxillary sinus. The incidence in males is 7 times that in females (85–88), with an average onset age of 60 years, and it is rare in children (89, 90). The clinical symptoms are not significantly specific. In the early stage, it is mainly characterized by unilateral nasal obstruction, epistaxis, and hyposmia. In the advanced stage, due to tumor invasion of the alveolus, orbit, and intracranial region, symptoms such as toothache, decreased vision, headache, and epilepsy may occur (87). CT shows an irregularly shaped soft tissue mass in the unilateral nasal cavity or sinus, with uniform density, and rare small cystic changes and calcification. There is obvious destruction of the sinus bone wall, nasal septum, pterygopalatine fossa, and skull base bone. MRI shows isointense or slightly hypointense signals on T1WI and slightly hyperintense signals on T2WI, with obvious heterogeneous enhancement on contrast-enhanced scanning (**Figure 11**). The imaging diagnosis lacks characteristic features and is easily misdiagnosed as olfactory neuroblastoma, squamous cell carcinoma, and undifferentiated carcinoma of the nasal cavity and paranasal sinuses (91). The definitive diagnosis mainly relies on pathology. The latest pathological research suggests that most teratoid carcinosarcomas are associated with SMARCA4 gene defects (92).

#### Lymphoma

Lymphoma is a common type of malignant tumors in the nasal cavity and paranasal sinuses, with diffuse large B-cell lymphoma (DLBCL) and NK/T-cell lymphoma (NKTCL) being the two most common subtypes (93). Both subtypes are more prevalent in males.

##### DLBCL

**Figure 12:**
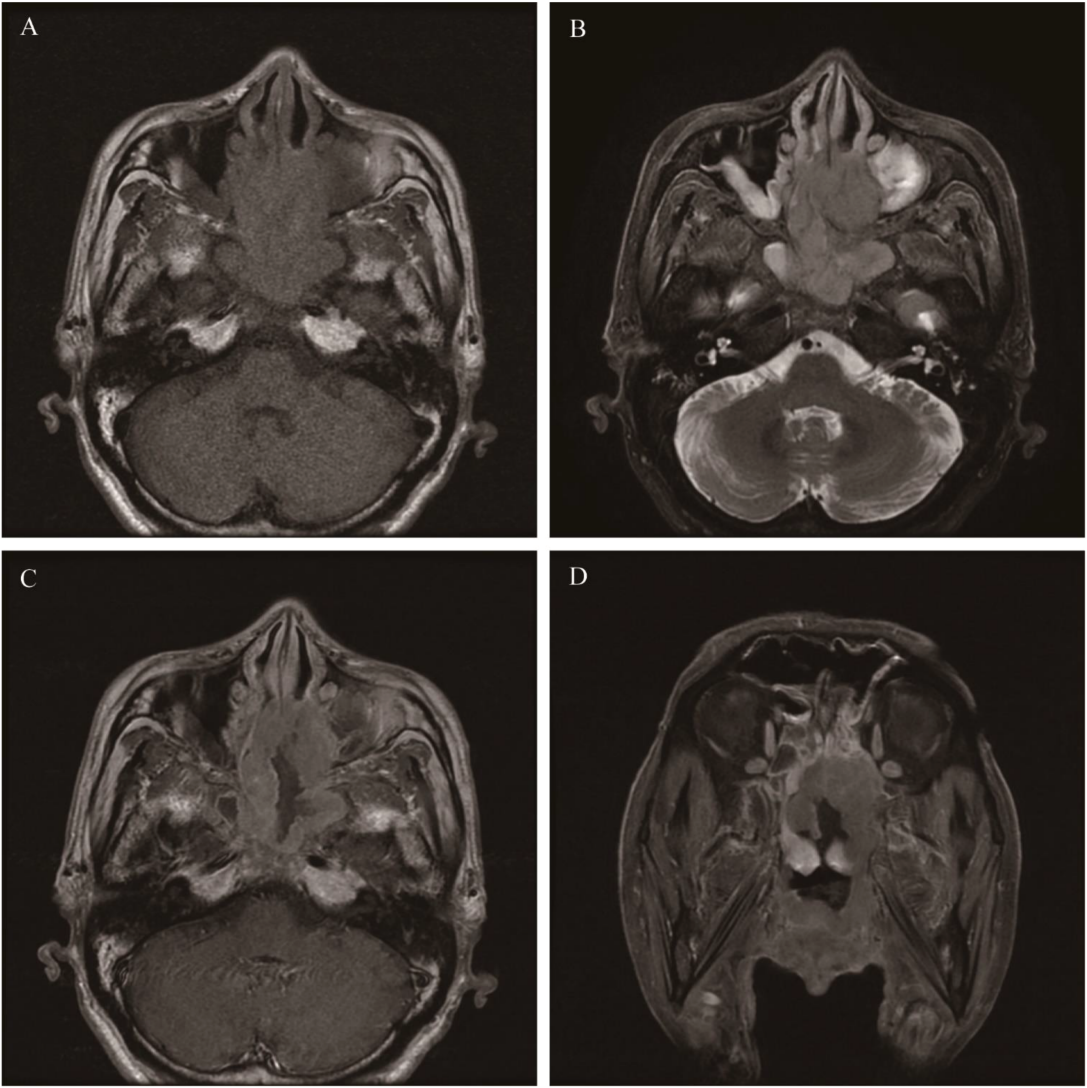
Diffuse large B-cell lymphoma: On T1WI, there was an irregular but homogeneous-signal mass located in the ethmoid sinus and sphenoid sinus, and the mass showed an isointense signal **(A)**. The mass appeared slightly hyperintense on T2WI **(B)**. Axial and coronal MR enhancement showed moderate enhancement, and the degree of enhancement is lower than that of the mucosa **(C, D)**.

The most common NHL subtype in adults is more prevalent in middle-aged and elderly individuals, with an average age of approximately 67.8 years (93). Lesions are mainly located in the sinuses, most commonly in the maxillary sinus, and unilateral lesions are more frequent than bilateral ones. Therefore, the common clinical symptoms are mainly nasal congestion and epistaxis. Cervical lymph node enlargement and fever are also common clinical symptoms. Proptosis and diplopia indicate lesion progression and orbital involvement. CT findings: Irregular soft-tissue masses in the nasal cavity with unclear boundaries and uniform density. Even if the tumor is large, liquefactive necrosis rarely occurs. Bone changes mainly include compressive displacement, bone resorption, and bone remodeling. Invasive bone destruction is rare, and its degree and scope are less severe than those of SCC. CT enhancement shows mild to moderate enhancement. MRI: T1WI shows an isointense or slightly higher signal than muscle, and T2WI shows a slightly high signal. The ADC value is lower than that of SCC, and it increases significantly after chemotherapy (**Figure 12**).

##### ENKTCL

**Figure 13:**
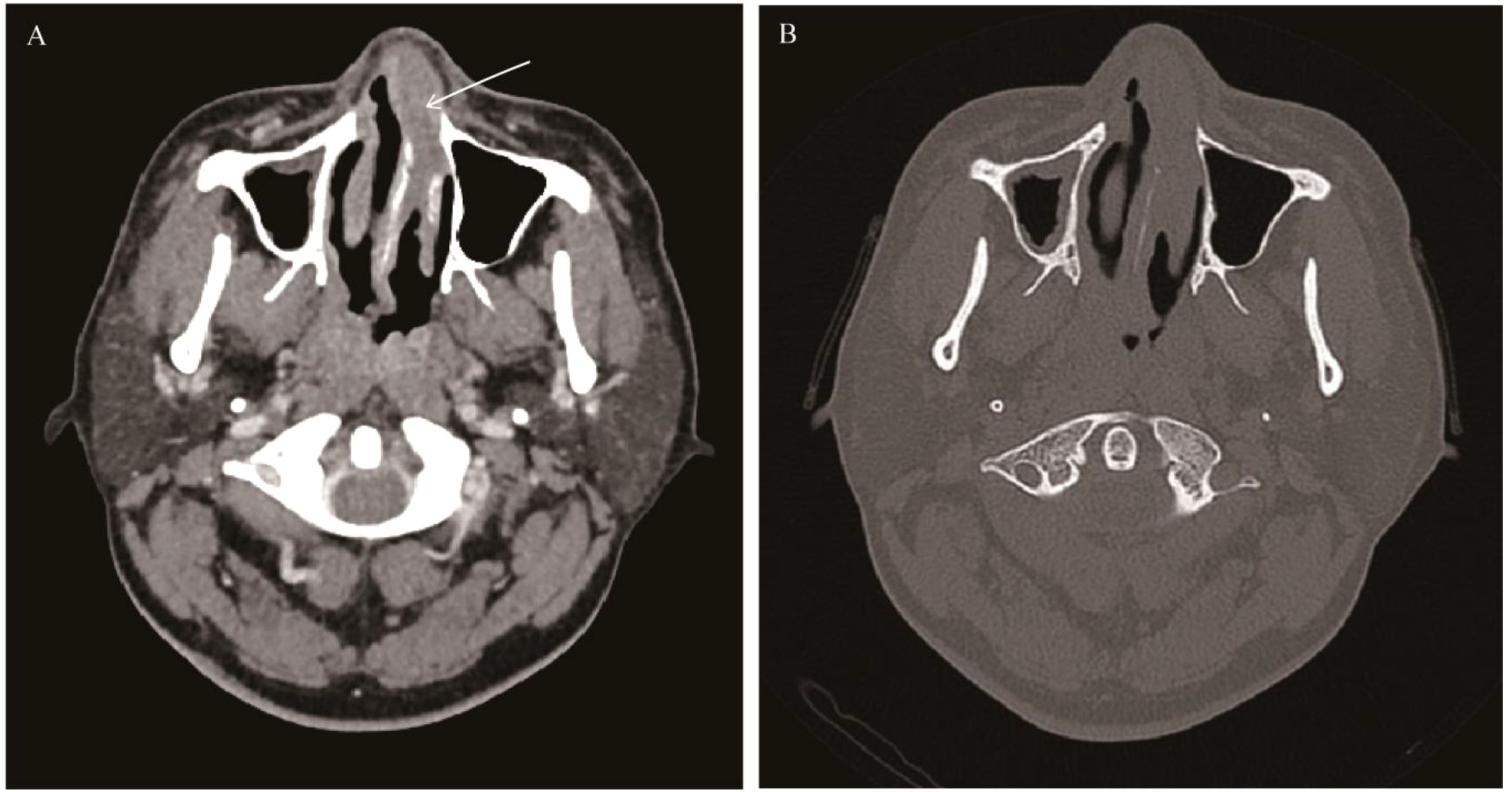
NK/T-cell lymphoma: Axial CT showed soft tissue masse in the left nasal cavity and nasal vestibule (white arrow), with mild bone destruction and uniform enhancement of the masse **(A, B)**.

Patients with ENKTCL are younger than those with DLBCL (94), with a median age of 40–50 years. The tumors are generally located in the nasal cavity and nasal vestibule, with unilateral lesions being more common. They tend to infiltrate along the submucosa and dermal lymphatic vessels, leading to the common clinical manifestations of thickening of the nasal dorsum, bilateral nasal alae, and facial skin. Under nasal endoscopy, mucosal swelling, extensive ulcers, and superficial necrosis can be observed (95). When patients first seek medical advice, the lesion volume is often smaller than that of DLBCL, all of which are characteristic. CT shows small, irregular soft-tissue masses, mostly located in the inferior turbinate, anterior part of the nasal septum, and nasal vestibule. They grow invasively and involve the external nose and mid-facial skin, resulting in the disappearance of the subcutaneous fat space in the nose and face. Due to liquefaction and cystic change, the density is inhomogeneous. CT enhancement is mainly mild-to-moderate inhomogeneous enhancement. MRI: T1WI shows isointense or slightly hypointense signals, similar to muscle. T2WI shows slightly hyperintense signals, with the signal intensity between that of muscle and mucosa. Since necrosis, hemorrhage, and cystic change are more common than in DLBCL, the signals are heterogeneous. MRI enhancement is similar to CT enhancement, and dynamic enhancement shows a progressive enhancement pattern (**Figure 13**).

#### Sinonasal metastasis

**Figure 14:**
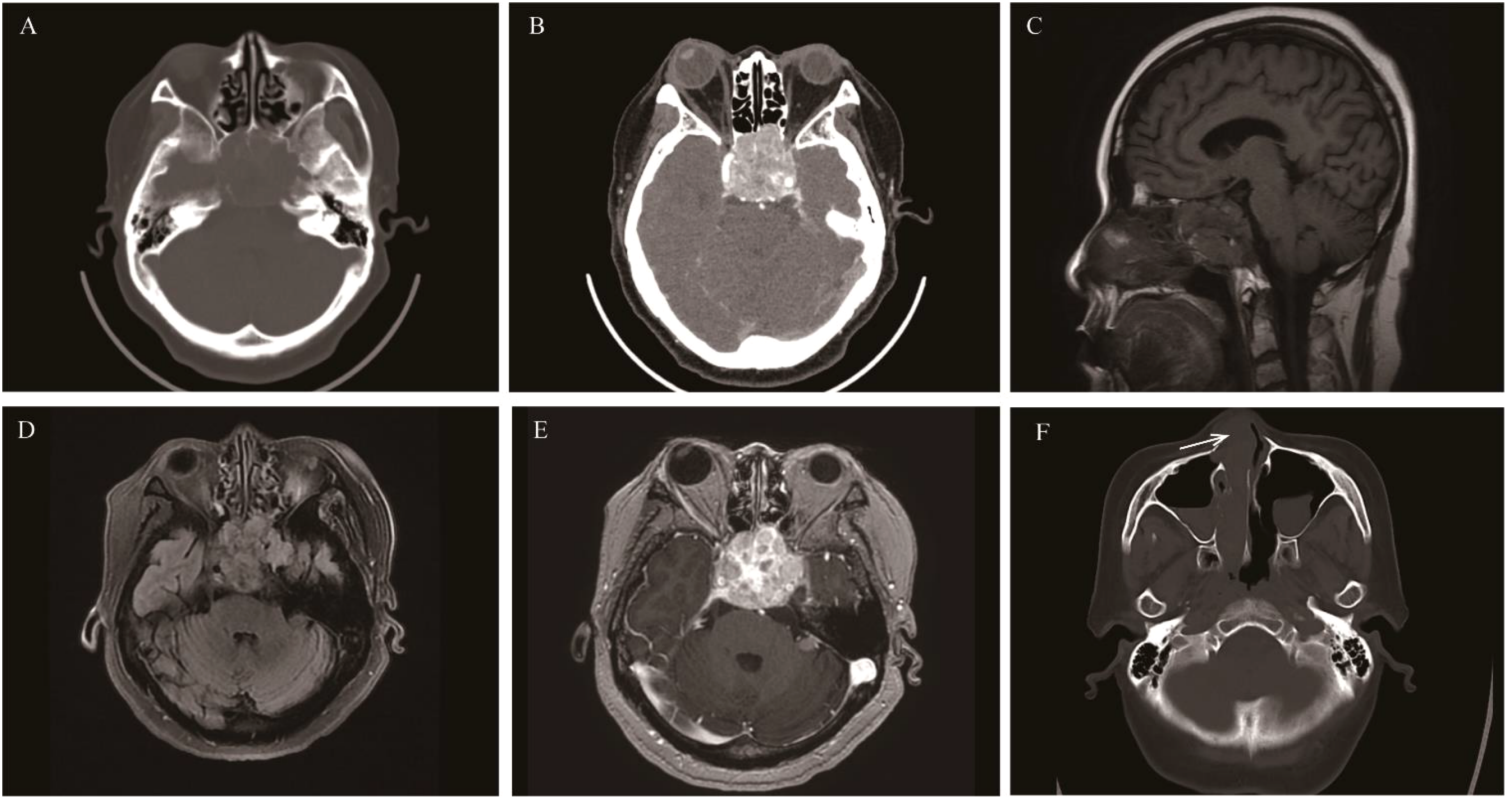
Sinonasal metastasis: Patient 1: A soft tissue mass involving the upper part of the nasal cavity, sphenoid sinus, ethmoid sinus, cavernous sinus, and clivus, with obvious bone destruction on CT **(A, B)**. It showed heterogeneous isointensity on T1WI **(C)**, hyperintensity on T2WI **(D)**, and marked enhancement on contrast-enhanced MRI **(E)**. Pathology confirmed that it was derived from clear cell renal carcinoma. Patient 2: A mass in the right nasal vestibule (white arrow), which was pathologically confirmed to be derived from hepatocellular carcinoma **(F)**.

Metastatic tumors of the nasal cavity and paranasal sinuses are particularly rare, and the literature mainly focuses on case reports. The primary tumors reported in the literature mainly originate from the lungs, liver, kidneys, thyroid, prostate, breast, and colon (96–100), with renal cancer metastases being the most common. The most likely metastatic route is hematogenous metastasis (101). It is speculated that tumor cells reach the head and neck via the systemic circulation, pass through the pterygoid plexus and cavernous sinus, and move retrogradely to the nasal cavity and paranasal sinuses. Due to the slow blood flow here, tumor cells can easily detach from the circulation and implant, leading to the growth of metastatic tumors. The speculated metastatic routes of metastases from the liver and lungs may be related to the paravertebral venous plexus and thoracic duct (102). Metastatic tumors of the nasal cavity and paranasal sinuses are generally located in the maxillary sinus. The clinical symptoms lack specificity, typically presenting as facial pain, recurrent epistaxis, and nasal obstruction. The imaging findings also lack specificity, usually showing single or multiple lesions. On CT, they appear as soft-tissue masses with obvious bone destruction, and large-sized tumors may have necrosis, cystic changes, or hemorrhage. On MRI, the T2WI signal is mostly heterogeneous. Due to the rich blood supply of metastatic tumors, they show moderate to marked heterogeneous enhancement, and DCE- MRI indicates the characteristic of high tumor perfusion. Some researchers have summarized that for middle-aged and elderly patients presenting with nasal obstruction or epistaxis, if imaging reveals isolated or multifocal, hypervascular lesions centered on bone destruction in the nasal cavity and paranasal sinuses, combined with the clinical history, metastatic tumors should be included in the common diagnoses (103) (**Figure 14**).

#### Osteosarcoma

**Figure 15:**
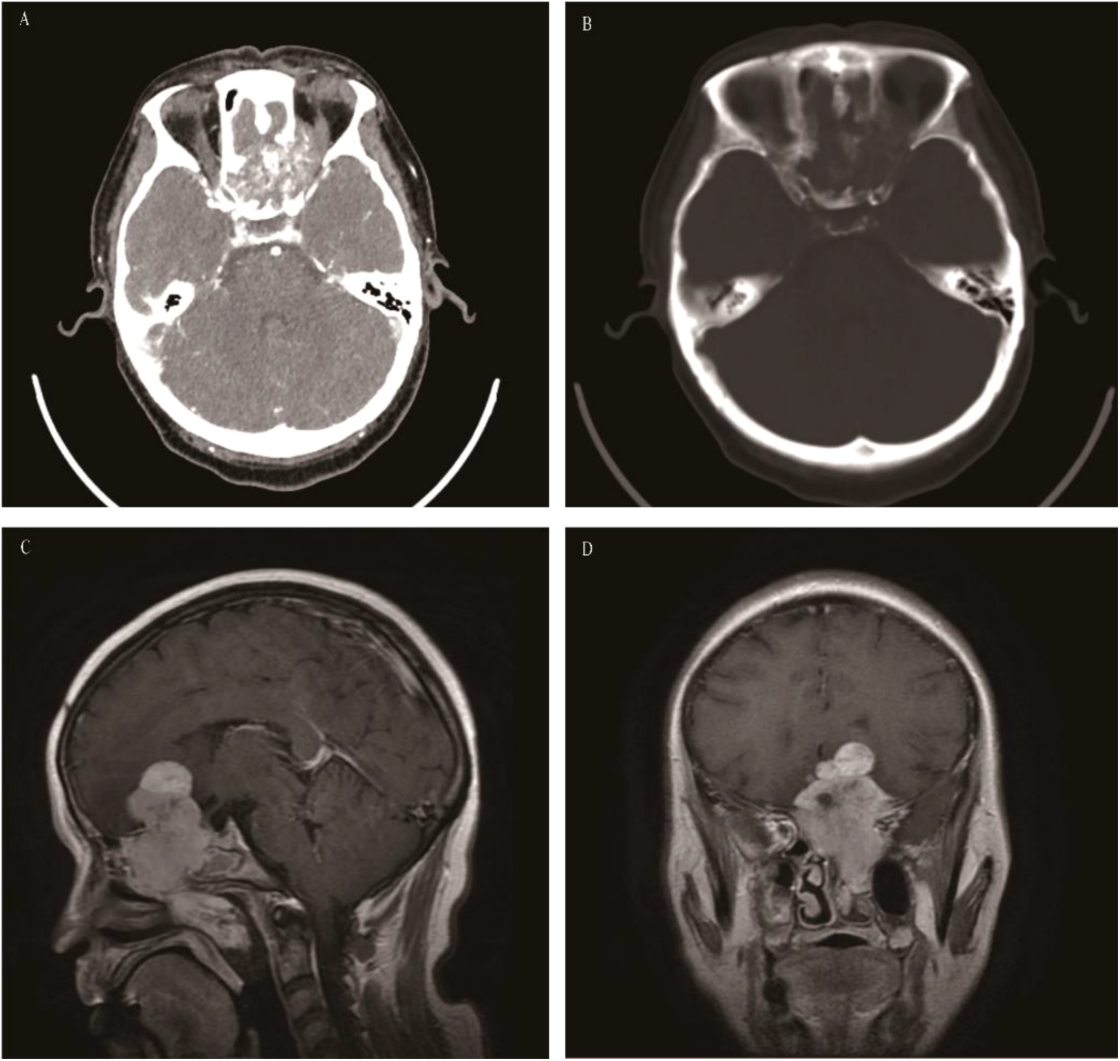
Osteosarcoma: Axial CT showed a mass in the left ethmoid sinus, which involved the left orbit and the intracranial region. Tumor bone was seen inside the mass, and there was obvious enhancement on contrast-enhanced CT **(A, B)**. Sagittal and coronal contrast-enhanced MRI showed obvious enhancement, and the tumor bone within the tumor presented as low signal **(C, D)**.

Osteosarcoma most commonly occurs in the metaphysis of long bones. Osteosarcoma located in the head and neck is rare, accounting for 15% of all osteosarcomas in the body. It mainly occurs in the mandible, followed by the maxilla, and is even rarer in the nasal cavity and paranasal sinuses (104). The median age of onset is 40 years (105). The clinical manifestations are non-specific and are often related to the site of onset. Since most lesions are located in the maxilla and mandible, the common symptoms are mostly facial masses, loose or fallen teeth. When the mass involves the nasal cavity and paranasal sinuses, epistaxis and nasal obstruction occur on this basis. There is an elevation of alkaline phosphatase in serology. The imaging manifestations are similar to those of long-bone osteosarcoma, showing both osteogenic and osteolytic features. CT shows an irregular soft-tissue mass with unclear boundaries, bone destruction accompanied by periosteal reaction. The typical ivory, cotton-wool and radial tumor bones inside the mass highly suggest the diagnosis of osteosarcoma. Due to the diverse internal components of osteosarcoma, the MRI signals are also mixed and variable. However, the tumor bone and periosteal reaction with low signals on both T1WI and T2WI can also suggest the diagnosis (**Figure 15**).

## Prospects

### Multidisciplinary collaboration

In the future diagnosis and treatment of nasal cavity and paranasal sinus tumors, multidisciplinary participation should be emphasized, including radiology, otolaryngology, neurosurgery, pathology, and oncology departments, to formulate personalized diagnosis and treatment plans.

### Multimodal imaging

It involves the fusion, registration, and comparative analysis of images of the same lesion obtained from different imaging modalities. Multimodal imaging technology integrates anatomical, morphological, and functional information, and can even acquire metabolic and molecular information. Ultimately, it enables the localization, characterization, staging, intraoperative navigation, and follow-up of tumors, which is more accurate than traditional single-modality imaging. Currently applied technologies include PET-CT/MRI, ultrasound, dual-energy CT, and photon CT imaging (106–108). In the future research field of multimodal imaging, the focus will be on developing imaging devices with higher resolution and sensitivity, and addressing issues related to the accuracy, imaging efficiency, and stability of multimodal imaging fusion technology.

### Radiomics

Radiomics extracts data from CT, MRI, and PET images in a high-throughput manner, including quantitative features of multiple parameters such as first-order statistical features, spatial geometric features, texture features, and wavelet features. It enables the analysis of tumor heterogeneity and reveals the potential laws of lesions. Compared with the simple visual analysis of images in traditional imaging, radiomics utilizes the data mined from images, which can be combined with patients’ clinical data, laboratory tests, pathological data, genomes, or proteomes. Subsequently, quantitative analysis is performed to ultimately provide more, accurate, and detailed information about the tumor lesion itself and the surrounding microenvironment (109). Currently, a large number of experimental literatures focus on the preoperative prediction of tumor benignity and malignancy, pathological grading, molecular typing, gene and protein expression, occult lymph node metastasis, recurrence risk, and drug efficacy, etc. (110).

### Molecular imaging technology

It is an important branch in the field of precision diagnosis and treatment of tumors. It mainly detects the changes in genes, proteins, and the microenvironment of tumors in the ultra-early stage at the microscopic level, enabling the ultra-early diagnosis and treatment of tumors. In the future, molecular imaging technology will also be integrated into the decision support system throughout the entire diagnosis and treatment cycle, playing a role in pre-operative grading diagnosis, intra-operative navigation, efficacy evaluation, and post-operative follow-up. With the development of nanotechnology and multimodal imaging technology, tumor molecular imaging technology aims to achieve breakthroughs in finding highly sensitive targeted markers, constructing probes with high specificity, high stability, and high biocompatibility, establishing high-resolution imaging techniques, and developing efficient therapeutic functions. For example: 1. Arbitrarily combine and integrate multiple imaging technologies (radionuclide, CT/MRI, ultrasound, fluorescence, and photoacoustic imaging) into one type of probe to achieve comprehensive visualization of the macroscopic morphology and microscopic functions of tumors (111). 2. Develop single-or multi-targeted probes that are more sensitive and stable, responding specifically to tumor receptors and the tumor microenvironment, as well as probes that respond to both receptors and the microenvironment (112–115). 3. Develop integrated probes with both diagnostic and therapeutic functions (chemotherapy, hyperthermia, photodynamic therapy, sonodynamic therapy, immunotherapy, radionuclide therapy, and gene therapy), where the diagnostic and therapeutic functional modules can be easily combined to maximize the effectiveness of the probes. However, currently, such technologies are in the experimental stage, and more investment is needed for their full integration into clinical practice (116).

### Artificial intelligence

In the era of big data, there are a large number of cases for the same tumor, and the radiogenomics and radiomics data of each patient’s tumor form a huge database. Artificial intelligence technologies mainly including machine learning (ML) and deep learning (DL) have developed rapidly in CT and MRI image analysis. Currently, artificial intelligence technologies mainly empower radiomics, and the two have become important analytical tools in imaging medicine (117), which are mainly applied in the development of various prediction models (118), such as diagnostic assistance, treatment response prediction, and prognosis assessment. ML is applied in tumor feature extraction and analysis, improving the accuracy of tumor diagnosis, the safety of treatment, and the reliability of prognosis assessment. For example, Ramkumar et al. 39 found that MRI-based structural analysis could potentially distinguish sinus SCC from inverted papilloma (accuracy: 89.1%), and the results were comparable to the manual evaluation by neuroradiologists (P=0.0004) (119). Ranjbar et al. used CT-based texture analysis to classify the HPV status of oropharyngeal SCC (accuracy: 75.7%) (120). The advantages of DL lie in achieving automatic feature extraction, reducing manual intervention, and identifying complex patterns and subtle changes in tumor images, such as internal texture changes and metabolic features of tumors (121, 122). Moreover, DL can be applied in multi-center studies and promote data sharing. With the continuous development of artificial intelligence algorithms and computing power, some algorithms can be used for the rapid and repetitive diagnosis and treatment of nasal and sinus tumors. In addition, artificial intelligence can also enhance multimodal imaging technology and molecular imaging technology, making image recognition and segmentation and prediction model construction more accurate and perfect. In the future, the development direction of AI combined with multimodal imaging, radiomics, and molecular imaging technology should focus on data standardization and sharing, multidisciplinary integration, and clinical implementation and promotion.
